# Matrix-bound nanovesicles isolated from decellularized tumors as platforms for targeting parent tumor cells and tumor-associated stromal cells

**DOI:** 10.1016/j.mtbio.2025.102355

**Published:** 2025-09-24

**Authors:** Zheng-Hong Chen, Ye-Rong Hu, Xing-Bo Yue, Kun Zhao, Huan Yang, Zhi-Gang Liu, Rui Xu, Wei-Dong Lü

**Affiliations:** a Department of Integrated Chinese and Western Medicine, Tumor Hospital of Shaanxi Province, Affiliated to the Medical College of Xi'an Jiaotong University, Xi'an, Shaanxi, 710061, China; b Department of Cardiovascular Surgery, The Second Xiangya Hospital of Central South University, Changsha, Hunan, 410000, China; c Department of Thoracic Surgery, Tumor Hospital of Shaanxi Province, Affiliated to the Medical College of Xi'an Jiaotong University, Xi'an, Shaanxi, 710061, China; d Department of Surgery, The Second Affiliated Hospital of Shaanxi University of Chinese Medicine, Xianyang, Shaanxi, 712000, China; e Department of Oncology, Tumor Hospital of Shaanxi Province, Affiliated to the Medical College of Xi'an Jiaotong University, Xi'an, Shaanxi, 710061, China

**Keywords:** Extracellular vesicles, Matrix-bound nanovesicles, Extracellular matrix, Cancer-associated fibroblasts, Tumor-associated macrophages

## Abstract

Matrix-bound nanovesicles (MBVs) are an emerging class of extracellular vesicles (EVs) that are integrated into the extracellular matrix (ECM). Tumor ECM-derived MBVs hold promise as platforms for targeted delivery of therapeutic agents to both parental tumor cells and surrounding stromal cells. In this study, a subcutaneous tumor model was established by implanting A549 human lung adenocarcinoma cells into immunodeficient mice. The mixed method was used to decellularize the tumor tissue, producing an ECM scaffold free of cellular components. Subsequently, MBVs were successfully isolated from the ECM of the decellularized tumors. Compared with tumor cell-derived liquid-phase EVs, acellular tumor MBVs were smaller in size and were demonstrated to transport proteins related to focal adhesion and protein binding. The in vitro binding affinity assays and cell culture experiments involving acellular tumor MBVs showed specific targeting affinity for ECM components, tumor cells, and tumor-associated stromal cells, including cancer-associated fibroblasts and tumor-associated macrophages. After loading of the drug doxorubicin, this platform selectively inhibited tumor cells and tumor-associated stromal cells both in vitro and in vivo. These results provide important insights for future research on the potential role of tumor ECM-derived MBVs in targeted cancer therapy and the modulation of premetastatic niches.

## Highlights

• Matrix-bound nanovesicles (MBVs) were effectively isolated from the extracellular matrix (ECM) of decellularized tumors.
• These tumor ECM-derived MBVs strongly bind to the tumor ECM, tumor cells, and tumor-associated stromal cells.
• These MBVs loaded with doxorubicin demonstrate both imaging capabilities and antitumor efficacy in tumor-bearing mice.
• These MBVs transport proteins that help them home to and maintain affinity with their parent cells.

## Graphical abstract

**Figure:**
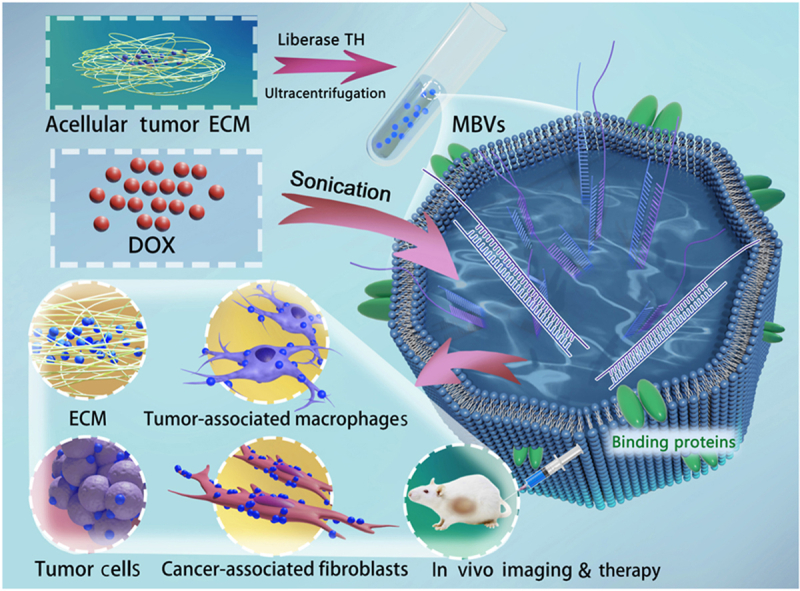

## **Abbreviations**

MBVs: matrix-bound nanovesicles
EVs: extracellular vesicles
ECM: extracellular matrix
MSCs: mesenchymal stem cells
TAMs: tumor-associated macrophages
CAFs: cancer-associated fibroblasts
PAA: peroxyacetic acid
DOX: doxorubicin
SCID: severe combined immunodeficiency
DMEM: Dulbecco's modified Eagle's medium
H&E: hematoxylin and eosin
GAGs: glycosaminoglycans
TEM: transmission electron microscopy
NTA: nanoparticle tracking analysis
SEM: scanning electron microscopy
FDA: fluorescein diacetate
FAP: fibroblast activation protein
LPS: lipopolysaccharide
IFN-γ, interferon-gamma
IL-6: interleukin-6
LIF: leukaemia inhibitory factor
TMT: tandem mass tag
RP-HPLC: reversed-phase high-performance liquid chromatography
DEP: differentially expressed protein
PCA: principal component analysis
GO: gene ontology
KEGG: Kyoto Encyclopedia of Genes and Genomes
PPI: protein‒protein interaction

## 1 Introduction

Solid tumors are the leading cause of cancer-related mortality. Solid tumor tissues consist of not only malignant cells but also nonmalignant host cells, including fibroblasts, immune cells, and endothelial cells, as well as structural components of the extracellular matrix (ECM) [1]. Within these tumor tissues, the interplay between cellular and noncellular components contributes to the formation of a tumor-promoting microenvironment [2,3]. However, treatment of solid tumors is difficult because of the complex tumor environment and drug resistance, which lead to poor drug targeting and major side effects [4]. Targeted therapy serves as a strategy to circumvent drug resistance and minimize adverse effects [5]. Extracellular vesicles (EVs) are secreted vesicles that mimic cell membranes, carry biomarkers for disease diagnosis and prognosis, and can evade immune detection and be well tolerated by the body [6]. They enable intercellular communication, can be stored long term without losing function, and are nonimmunogenic, making them promising candidates for promoting tissue repair after tumor surgery. EVs from cells outperform synthetic nanocarriers in drug delivery because of their superior biocompatibility and improved target specificity. They offer benefits such as deep tissue penetration and an extended circulation time. Additionally, their membrane components, including phospholipids and signalling factors, help them cross biological barriers such as the blood‒brain barrier [7]. EVs derived from tumor cells [8], mesenchymal stem cells (MSCs) [9], tumor-associated macrophages (TAMs) [10], cancer-associated fibroblasts (CAFs) [11,12], and tumor tissues [13,14] have been used in the diagnosis and treatment of cancer.

In natural biological systems, EVs are secreted by various cell types, and their functions depend on their parent cell type [15]. Recent research has identified a subset of EVs embedded within the matrix fibres of the ECM in soft tissues, termed matrix-bound nanovesicles (MBVs) [16]. MBVs are a unique type of EV that are 20 to 400 nm in size, are closely linked to the ECM and are not easily separated via standard decellularization methods. They contain bioactive molecules such as proteins, lipids, nucleic acids, and cytokines that affect cellular behavior [17]. They are distinct from exosomes in terms of both lipid membrane composition and molecular content, and they exhibit reduced or negative expression of specific exosome markers, such as CD9, CD63, and CD81 [18,19]. MBVs have been investigated extensively in the context of regenerative medicine. Notably, MBVs derived from gingival mesenchymal stem cells have been demonstrated to increase the proliferation of tracheal precursor cells and basal epithelial cells [20]. Furthermore, synthetic MBV complexes have been employed in research on regenerative medicine [21]. With respect to their influence on inflammatory cells and factors, MBVs sourced from marine organisms have been shown to inhibit the production of inflammatory cytokines [22]. MBVs derived from the bladder stroma and small intestinal submucosa have been shown to induce a phenotypic shift towards the M2 subtype in macrophages, which facilitates tissue regeneration [23]. In 3D culture, human bone marrow mesenchymal stem cells produce ECM, and MBVs extracted from that ECM increase fibroblast growth, shift macrophages to an anti-inflammatory state, and aid in the recovery of human forebrain organoids after starvation [24]. Moreover, breast-derived MBVs from older individuals are more likely to enhance breast cancer invasion than those from younger individuals are [25]. MBVs show no cytotoxicity in cell cultures, no toxicity in organisms, and no immunosuppressive effects, highlighting their potential for clinical use [26]. MBVs have potential for use in the design of platforms for targeted drug delivery to distal organs, as they carry select molecular cargoes. However, the biological functions of MBVs remain largely unexplored, and investigating MBVs will reveal novel functional roles and transfer mechanisms associated with EVs.

The ECM of tumors has unique attributes in terms of composition, structure, and biomechanics, which contribute to a specific architecture and set of biological functions that facilitate tumorigenesis and cancer progression [27,28]. Decellularization involves the removal of native cells and genetic material from the ECM, while keeping its structural, biochemical, and biomechanical features intact [29]. Scientists have begun exploring the use of tumor decellularization to create an ECM that preserves the mechanical traits and structure of tumors [27,30]. This decellularized ECM can be used in tissue-engineered models to closely mimic real tumors. The use of decellularized tumors in research has highlighted potential therapeutic targets and revealed mechanisms of cancer development and responses to chemotherapy [31]. The tumorigenic ECM is produced by both CAFs and tumor cells [32]. In our previous study, A549 human pulmonary adenocarcinoma cells were used to generate subcutaneous tumors in immunodeficient mice, and a multistep treatment was used to obtain a tumor ECM scaffold with complete removal of cellular components; the decellularized tumor ECM was used as a suitable 3D scaffold for tumor growth and antitumor drug sensitivity testing [33,34].

In this study, a subcutaneous tumor model was established with A549 human lung adenocarcinoma cells implanted in immunodeficient mice, and a modification of the mixed method involving Tris-Triton-peroxyacetic acid (PAA) was used to decellularize native tumor tissue, resulting in a tumor ECM scaffold with complete removal of cellular components. We next successfully isolated and extracted MBVs from decellularized tumor ECM and studied their targeting affinity for tumor cells and the tumor microenvironment, including the tumor ECM and stromal cells. After loading of the drug doxorubicin (MBV-DOX), we investigated the targeted inhibitory effect of the developed platform on tumor cells and tumor-associated stromal cells, such as CAFs and TAMs. Moreover, a tumor-bearing mouse model was used to evaluate the affinity and antitumor effects of MBV-DOX. Finally, quantitative proteomics analysis of acellular tumor MBVs in comparison to liquid-phase EVs was performed. We hypothesize that acellular tumor MBVs target tumors and their microenvironment with high affinity, possibly because the MBVs contain protein components involved in homing to tumors and their stroma.

## 2 Materials and methods

### 2.1 Preparation and assessment of acellular tumor MBVs

#### 2.1.1 Preparation and evaluation of ECM from decellularized tumors

**Development of a subcutaneous tumor model in SCID mice:** Following thawing, human lung adenocarcinoma A549 cells were incubated in Dulbecco's modified Eagle's medium (DMEM) supplemented with 1 % penicillin‒streptomycin and 10 % foetal bovine serum (FBS) under an atmosphere with 5 % CO2 at 37 °C. The cultured cells were subsequently digested with trypsin, resuspended in culture medium, and injected (3 × 10^5^ cells) into the axillary region of 6-week-old male severe combined immunodeficiency (SCID) mice (acquired from the Experimental Animal Center of Xi'an Jiaotong University). Approximately 30 days post-implantation, tumors that had reached a diameter of approximately 1 cm were excised, sectioned into thin slices, and further divided into smaller pieces for subsequent experimental procedures.

**Decellularization treatment:** The decellularization process adhered to a previously established methodology [33], with some modifications. Briefly, the decellularization protocol started with the immersion of excised tumor tissue in a low osmotic Tris buffer solution (10 mM Tris, 5 mM EDTA, pH 8.0) at 0 °C overnight. This was followed by a 24-h incubation period in a high osmotic Tris buffer solution (50 mM Tris, 1 M NaCl, 10 mM EDTA, pH 8.0) at 37 °C. The cellular components were subsequently extracted by treating the tissue with 0.5 % (v/v) Triton X-100 and 0.02 % EDTA for 6 h and then incubating it in 0.1 % (v/v) peroxyacetic acid and 4 % (v/v) ethanol for 2 h. The entire decellularization procedure was conducted on a shaker set at 70 rpm at a temperature of 37 °C, and at the end of the process, the samples were subjected to extensive rinsing with sterile phosphate-buffered saline (PBS).

**Evaluation of cell removal:** Tissue samples were fixed in 4 % formaldehyde for 24 h and subsequently embedded in paraffin. Following sectioning, standard hematoxylin and eosin (H&E) staining was conducted to assess the extent of cell removal. Masson's trichrome staining was used to visualize collagen fibres, whereas Scott's Alcian blue staining was used to visualize glycosaminoglycans (GAGs). Native and decellularized tumors were subjected to freeze-drying, after which DNA extraction was performed using a DNA extraction kit (Mo Bio Lab) with a sample size of eight (n = 8). The DNA content before and after decellularization was quantified using a PicoGreen dsDNA quantitation kit (Invitrogen) following previously established protocols [33].

#### 2.1.2 Preparation of acellular tumor MBVs

The effective separation and extraction of MBVs represents a pivotal technological contribution of this study. A prerequisite for this process is the ability to disrupt the ECM structure associated with MBVs while ensuring that the integrity of the MBVs is preserved. In this study, Liberase TH was employed to degrade the ECM enzymatically, which facilitated the extraction of MBVs via ultracentrifugation. Furthermore, since EVs derived from tumor cell culture medium were used as the control group, this study also involved the extraction and characterization of EVs from the tumor cell culture supernatant.

**EV extraction from the tumor cell culture supernatant:** Human lung adenocarcinoma A549 cells were cultured and propagated in exosome-free medium (Beyotime Biotech., Haimen, China). The supernatant from the culture medium was collected for subsequent use, and EVs were isolated via a sequential centrifugation process: initially at 500×*g* for 10 min, followed by 2,500×*g* for 20 min, and finally at 10,000×*g* for 30 min. This was followed by ultracentrifugation at 4 °C at 100,000×*g* for 2 h. The isolated EVs were then resuspended in sterile PBS, filtered through a 0.22-μm polyethersulfone (PES) filter (Millipore), and stored at −80 °C for future use.

**Separation and extraction of acellular tumor MBVs:** To facilitate the release and separation of MBVs, the decellularized tumor ECM was subjected to freeze-drying for 24 h, followed by pulverization into a fine powder using a Wiley mini mill equipped with a 60-mesh sieve (Arthur K. Thomas Co., Philadelphia, PA, USA). The ECM was subsequently dissolved in a 1.5 mL Eppendorf tube containing 1 mL of Liberase TH at a concentration of 0.01 mg/mL (Sigma Aldrich), in accordance with established protocols [35]. The enzyme mixture was prepared in 50 mM Tris buffer (pH 8) supplemented with 5 mM CaCl2 and 200 mM NaCl. The ratio of decellularized tumor ECM to enzyme release solution was maintained at 10 mg/mL to 1 mL. High-speed oscillation was conducted utilizing a vortex oscillator for a duration of 10 s. Furthermore, the mixture was incubated on a shaker at 25 °C for 12 h. To eliminate undissolved collagen and other components, a series of centrifugation steps were carried out, with each step performed in triplicate: initially at 500×*g* for 10 min, then at 2,500×*g* for 20 min, and finally at 10,000×*g* for 30 min. The supernatant was ultracentrifuged at 100,000×*g* for 2 h at 4 °C to isolate the MBVs, which were subsequently resuspended in sterile PBS. The solution was filtered with a 0.22-μm PES filter and preserved at −80 °C for future use.

#### 2.1.3 Evaluation of tumor MBVs

**Transmission electron microscopy (TEM):** Vesicles (1 × 10^9^ particles/mL) were deposited onto carbon-coated grids for 10 min and subsequently fixed with 4 % paraformaldehyde for 30 min. Following fixation, the grids were washed with distilled water and air-dried. The samples were then postfixed with 4 % osmium tetroxide for 10 min at room temperature. The samples were stained with 2 % uranyl acetate for 10 min at room temperature. Imaging was performed using a Talos L120C G2 transmission electron microscope (Thermo Fisher Scientific, Waltham, MA, USA) at a high voltage of 120 kV.

**Zeta potential assays:** Liquid-phase EVs and acellular tumor MBVs were diluted to a final concentration of 1 × 10^9^ particles/mL (n = 3). Zeta potential measurements were conducted utilizing a ZetaView Particle Analyzer (ZetaView PMX110, Japan). Each sample was analysed in triplicate, and the mean zeta potential value was derived from electrophoretic mobility measurements, with adjustments made for the viscosity and dielectric constant of the buffer. To evaluate the stability of liquid-phase EVs and acellular tumor MBVs, the samples were stored at 4 °C, and their zeta potentials were measured at intervals of 0, 6, 24, 72, 120, and 240 h.

**Nanoparticle tracking analysis (NTA):** NTA was employed to quantify and assess the size distributions of liquid-phase EVs and acellular tumor MBVs. The experimental procedures were conducted with a NanoSight NS300 instrument (Malvern Instruments Ltd., Malvern, UK), which facilitated the analysis of freshly resuspended MBVs or EVs in particle-free PBS. A sample volume of approximately 0.3 mL was introduced into a viewing chamber within a laser illumination module to measure beam refraction. The particle sizes and concentrations were determined from three 60-s video recordings, with data analysis performed using NTA3.2 Nanosight software.

**Western blot analysis:** Total protein was extracted utilizing an Invitrogen exosome protein extraction kit, and the protein concentration was quantified via a Bradford assay. Proteins were subsequently separated by SDS‒PAGE, following the protocol outlined in a previous study [36]. After blocking with nonfat milk, the membrane was incubated overnight at 4 °C with primary rabbit anti-mouse antibodies specific for CD9, CD81, Calnexin, and Alix, each diluted 1:1000 (Abcam). Finally, the membrane was incubated with a horseradish peroxidase-conjugated goat anti-rabbit secondary antibody (Invitrogen), and images were captured for further analysis.

**Hemolytic assays:** Blood samples were obtained from the orbital vein of healthy rats. Initially, approximately 0.5 mL of blood was collected and subjected to centrifugation at 5000 rpm for 10 min at 4 °C. The supernatant, which contained white blood cells and plasma, was discarded. The remaining red blood cells were washed five times with physiological saline and subsequently resuspended in physiological saline to prepare a 2 % red blood cell suspension. A specified quantity of material was then added to the 2 % cell suspension, with vesicle concentrations of 0.5, 1, 2, 5, 10, and 20 × 10^8^ particles/mL, respectively. Physiological saline and Triton X-100 were utilized as negative and positive controls, respectively. Following gentle vortexing, the samples were incubated at 37 °C for 3 h. After incubation, each sample was centrifuged at 5000 rpm for 15 min at 4 °C, and the supernatant was collected. A volume of 100 μL of the supernatant from both the control and experimental groups was transferred to a 96-well plate, and absorbance was measured at 450 nm using a microplate reader. The hemolysis rate was subsequently calculated using the appropriate formula: Hemolysis (%) = [(A_sample_ - A_negative control_)/(A_positive control_ - A_negative control_)] × 100 %.

### 2.2 In vitro binding affinity and cell culture experiments

#### 2.2.1 Investigation of the binding of acellular tumor MBVs to tumor ECM

Acellular tumor MBVs and control liquid-phase EVs were labelled with the lipophilic fluorescent dye PKH-26 (Sigma Aldrich) according to the manufacturer's protocol. In brief, the labelled EVs were resuspended in an appropriate volume of 1 × PBS and subjected to ultracentrifugation at 120,000×*g* for 70 min at 4 °C. After the supernatant was removed, the pellet was carefully resuspended in a suitable volume of 0.25 M glucose and again subjected to ultracentrifugation under the same conditions to obtain the final PKH26-EV preparation. Recognizing that EV labelling efficiency is typically below 100 % and that the washing process inevitably results in some loss of EVs, an initial EV quantity double that required for subsequent experiments was used for the labelling procedure. Furthermore, to minimize potential contamination, the prepared PKH26-EVs were filtered through a 0.22 μm membrane prior to use. The PKH-26-labelled acellular tumor MBVs and liquid-phase EVs, which were standardized to a protein concentration of 60 μg/mL, were coincubated with decellularized tumor ECM for a duration of 6 h. This was followed by three washes with sterile PBS. Following frozen sectioning at a slice thickness of 8 μm, the binding and fluorescence intensity of acellular tumor MBVs and control liquid-phase EVs with ECM were quantified and compared via fluorescence microscopy, and ImageJ software employed to evaluate their affinity for tumor ECM. Decellularized tumor ECM and ECM incubated with liquid-phase EVs and acellular tumor MBVs were subsequently fixed with a 0.3 % glutaraldehyde solution. The samples were then analysed by scanning electron microscopy (SEM) with a Hitachi S4800 instrument (Hitachi, Tokyo, Japan).

#### 2.2.2 Study of the affinity of acellular tumor MBVs for tumor cells and the impact of doxorubicin (DOX)-loaded MBVs on tumor cell growth

**Study of tumor cell affinity:** Human lung adenocarcinoma A549 cells were thawed and cultured. Acellular tumor MBVs and control liquid-phase EVs, which were both labelled with PKH-26, were incubated with A549 cells at a concentration of 2 × 10^5^ cells/mL for 6 and 24 h. Subsequently, live cells were stained with fluorescein diacetate (FDA) as described in previous studies [33,37]. The fluorescence intensities of PKH-26-labelled MBVs and EVs internalized by tumor cells, as well as FDA-labelled live cells, were observed via fluorescence microscopy and compared. The PKH-26 fluorescence intensity was quantitative analysis using ImageJ software.

**DOX loading procedure:** Acellular tumor MBVs and liquid-phase EVs (1 × 10^9^ particles, as quantified by NTA) were aliquoted into 500 μL of PBS and combined with 1 μg of DOX. The resulting mixture was sonicated with a Qsonica Q700 sonicator equipped with a 3.2 mm probe operating at 20 % amplitude with alternating 10-s on and off pulses for a total of three cycles. This was followed by incubation at 37 °C for 30 min to facilitate membrane reformation. Subsequently, DOX-loaded acellular tumor MBVs (MBV-DOX) and liquid-phase EVs (EV-DOX) were isolated via ultracentrifugation at 120,000×*g* for 90 min. Additionally, to remove unincorporated free DOX and isolate MBVs or EVs, the samples were subjected to washing with cold PBS, followed by a second ultracentrifugation step under identical conditions. The loading efficiency was determined by quantifying the UV/Vis absorbance of the unincorporated DOX present in the supernatant at a wavelength of 480 nm.

**In vitro drug release profiles:** The drug release profiles of EV-DOX and MBV-DOX were systematically examined over a 24-h duration at two distinct pH levels: 7.4, simulating the physiological blood environment, and 5.5, mimicking the endosomal environment. The experimental setup involved transferring the solutions into dialysis bags with a molecular weight cut-off (MWCO) of 3,500 Da, which were subsequently immersed in 50.0 mL of the respective buffer solutions maintained at 37 °C on an orbital shaker. Samples of 2.0 mL were extracted from the dialysis medium at specified intervals (2, 4, 6, 8, and 24 h) for analysis, with an equivalent volume of fresh buffer added to sustain a constant volume. The quantification of DOX released from EV-DOX or MBV-DOX was performed using high-performance liquid chromatography (HPLC).

**Evaluation of in vitro cytotoxic effects on normal cell lines:** The cytotoxic effects of MBVs and MBV-DOX on L929 mouse fibroblasts and mouse brain-derived endothelial cells (BEND3) were evaluated using the Cell Counting Kit-8 (CCK-8) assay. Initially, cells were seeded in a 96-well plate at a density of 1 × 10^4^ cells per well and incubated overnight. Subsequently, the cells were exposed to varying concentrations of DOX, encapsulated in EV-DOX and MBV-DOX, at 0.125, 0.25, 0.5, 1.0, 2.0, 4.0, and 8.0 μM. Additionally, treatments included a PBS control, liquid-phase EVs, and MBVs at a concentration of 1 × 10^9^ particles/mL, as well as DOX, EV-DOX, and MBV-DOX at a fixed DOX concentration of 1.0 μM. Following a 24-h incubation period, cell viability was assessed using the CCK-8 assay (Beyotime, Jiangsu, China). The results were expressed as relative cell viability, normalized to the viability of cells treated with PBS.

**Incubation of DOX-loaded MBVs with A549 cells:** Free DOX, MBV-DOX or EV-DOX (DOX concentration of 1 μM) were incubated with A549 cells (concentration of 2 × 10^5^ cells/mL) in DMEM containing 1 % penicillin streptomycin and 10 % FBS at 37 °C and 5 % CO2 for 24 h. After the culture medium was replaced, merged DOX autofluorescence and light microscopy images of A549 cells were obtained. Quantification of the DOX encapsulated within the EVs was conducted via fluorescence intensity measurements with a Synergy microplate reader (Biotek).

**Measurement of cell viability:** A549 cells were cultured in a 96-well plate at a seeding density of 12,000 cells per well and incubated for 72 h. Viable cells were subsequently stained with FDA following the protocol outlined in a previous study [33]. The cytotoxic effects of free DOX, EV-DOX or MBV-DOX, each at a DOX concentration of 1 μM, on A549 cells were assessed utilizing the alamarBlue assay, as detailed in a previous study [38].

#### 2.2.3 Research on the affinity of acellular tumor MBVs for fibroblasts and CAFs and the effect of DOX-loaded MBVs on cell growth

**Isolation and characterization of fibroblasts and CAFs:** CAFs were isolated in accordance with established protocols [39]. In brief, tumor tissue blocks were obtained via surgical resection of subcutaneous tumors in mice. These blocks were then washed with sterile PBS and transferred to a sterile work surface. The tissue blocks were subsequently dissected into smaller fragments using sterile forceps and scissors. The dissected tissue fragments were collected in a 15 mL centrifuge tube containing DMEM supplemented with 0.2 % trypsin, 0.2 % collagenase A, and 5 % FBS. The tube was incubated for 4 h to facilitate digestion, after which the supernatant was centrifuged at 1,300 rpm for 4 min. The supernatant was subsequently removed, and the cells were resuspended in DMEM supplemented with 1 % penicillin‒streptomycin and 10 % FBS for culture. Once the cells had fully adhered to the substrate and proliferated, they were subjected to enzymatic digestion and transferred to a new culture dish. The cells were then purified and isolated via the adhesion method. After three passages, purified CAFs were obtained for identification and further experimentation. The control fibroblasts were obtained by isolating and culturing subcutaneous fibroblasts from mice that had not been subjected to tumor implantation, following the same protocol used for CAF isolation. Fibroblast activation protein (FAP), classified as a type II membrane serine protease, is widely recognized as a prototypical marker for CAFs [40]. The overexpression of FAP on activated fibroblasts is associated with adverse prognosis and outcomes in various cancers, highlighting the potential of FAP-targeted imaging and therapeutic strategies [41]. To distinguish fibroblasts from CAFs, double immunofluorescence staining was performed using goat anti-vimentin antibodies (dilution 1:200, Invitrogen) and rabbit anti-FAP monoclonal antibodies (dilution 1:200, Invitrogen). The secondary antibodies, namely, Alexa Fluor 647-conjugated donkey anti-goat IgG and Alexa Fluor 488-conjugated goat anti-rabbit IgG, were obtained from Abcam. The cell nuclei were counterstained with DAPI. The detailed protocol for immunofluorescence staining has been described previously [34,37].

**Investigation of the affinity of acellular tumor MBVs for fibroblasts and CAFs and the effects of DOX-loaded MBVs on cell growth:** PKH-26-labelled acellular tumor MBVs and control liquid-phase EVs were incubated with fibroblasts or CAFs at a concentration of 2 × 10^5^ cells/mL for 6 and 24 h. Subsequently, live cells were stained with FDA. Dual staining with PKH-26 and FDA was visualized via fluorescence microscopy. To determine the order of PKH-26-labelled vesicles appearance in the cell membrane, nucleus, and cytoplasm for CAFs, Hoechst 33342 was used at different time points of uptake (2, 4, 6, and 24 h). Additionally, free DOX, EV-DOX or MBV-DOX were incubated with fibroblasts or CAFs at the same concentration for 24 h. Merged DOX autofluorescence and light microscopy images were analysed for both fibroblasts and CAFs. The fluorescence intensity of the loaded DOX was quantitated. Finally, live cells were stained with FDA, and cell viability measurements were performed after fibroblasts or CAFs (12,000 cells/well) were incubated with free DOX, EV-DOX or MBV-DOX for 72 h.

#### 2.2.4 Evaluation of the affinity of acellular tumor MBVs for macrophages and the effect of DOX-loaded MBVs on macrophage growth

Upon activation, macrophages can be categorized into two distinct phenotypes: classically activated macrophages (M1 macrophages) and alternatively activated macrophages (M2 macrophages). M1 macrophages are characterized by their tumor-inhibitory functions, whereas M2 macrophages are associated with the promotion of tumor growth [42]. Within the tumor microenvironment, tumor-associated macrophages (TAMs) are predominantly the M2d subtype of M2 macrophages [43].

**Induction of M1 and M2d macrophages:** Mouse bone marrow-derived macrophages (M0) were obtained from ScienCell (catalog number M1920). After thawing, the macrophages were cultured in a sterile 6-well plate with macrophage culture medium (ScienCell, M1921). The cells were incubated under an atmosphere with 5 % CO2 at 37 °C for three days. The cells were subsequently digested with 0.25 % trypsin and washed with PBS, and their concentration was adjusted to 1.5 × 10^6^ cells/mL for further use. M0 macrophages were converted to M1 macrophages using 100 ng/mL lipopolysaccharide (LPS) and 20 ng/mL interferon-gamma (IFN-γ) and converted to M2d macrophages using 50 ng/mL interleukin-6 (IL-6) and 25 ng/mL leukaemia inhibitory factor (LIF), followed by a 24-h incubation, as outlined previously [44]. To confirm macrophage polarization, double immunofluorescence staining was performed using rat anti-F4/80 (dilution 1:50, Invitrogen) and rabbit anti-CD86 (dilution 1:200, Invitrogen) antibodies for M1 macrophages and rat anti-F4/80 (dilution 1:50, Invitrogen) and rabbit anti-vascular endothelial growth factor A (VEGF-A) (dilution 1:200, Invitrogen) antibodies for M2d macrophages. The Alexa Fluor 647-conjugated donkey anti-rat IgG and Alexa Fluor 488-conjugated goat anti-rabbit IgG secondary antibodies were from Abcam, along with DAPI for nuclear staining.

**Investigation of the affinity of acellular tumor MBVs for M1 and M2d macrophages and the effects of DOX-loaded MBVs on macrophage growth:** PKH-26-labelled acellular tumor MBVs and control liquid-phase EVs were incubated with M1 or M2d macrophages at a concentration of 2 × 10^5^ cells/mL for 6 and 24 h. Live cells were subsequently stained with FDA. Dual staining with PKH-26 and FDA was evaluated via fluorescence microscopy. Additionally, free DOX, EV-DOX or MBV-DOX were incubated with M1 or M2d macrophages at the same concentration for 24 h. The merged DOX autofluorescence and light microscopy images were analysed, and the fluorescence intensity of the loaded DOX was quantified. Live cells were stained with FDA, and cell viability was assessed following a 72-h incubation of M1 or M2d macrophages (12,000 cells per well) with free DOX, EV-DOX or MBV-DOX.

### 2.3 Animal experiments

#### 2.3.1 Animal treatment

All animal care and handling procedures were conducted in accordance with national guidelines and approved by the Animal Research Committee of Xi'an Jiaotong University (No. XJTUAE20241185). SCID mice aged 4–5 weeks were obtained from the Animal Centre of Xi'an Jiaotong University. The skin on the right side of the back of each mouse was sterilized with 75 % ethanol. A suspension of A549 tumor cells (100 μL, containing 3 × 10^5^ cells in 100 μL of PBS) was administered subcutaneously into the designated region. When the transplanted tumor reached an approximate volume of 20 mm^3^, the mice were subjected to intravenous treatment with 100 μL of various formulations, including free DOX, EV-DOX, and MBV-DOX, each at a DOX concentration of 5 mg/kg, and a PBS solution served as the negative control.

#### 2.3.2 Pharmacokinetics

To investigate the pharmacokinetics, female SCID mice were administered 100 μL of various formulations, including free DOX, EV-DOX, and MBV-DOX, each at a DOX concentration of 5 mg/kg. Blood samples were collected from the retro-orbital sinus at intervals of 5, 10, 15, and 30 min, as well as 1, 3, 12, 24, 48, and 72 h post-injection. The collected blood was immediately transferred into heparin-coated tubes to prevent coagulation. Subsequently, the samples were centrifuged at 3000 g for 10 min to isolate the plasma. The concentration of DOX in the plasma was quantified using HPLC, and concentration-time curves were generated. Pharmacokinetic parameters were then calculated and analysed using DAS2.0 software.

#### 2.3.3 In vivo and ex vivo imaging

SCID mice bearing tumors with an approximate volume of 200 mm^3^ were used to investigate the in vivo biodistribution and targeting efficacy of DOX-loaded acellular tumor MBVs. Briefly, 100 μL of each formulation (PBS, free DOX, EV-DOX, or MBV-DOX) was administered intravenously via the tail vein. The fluorescence signal intensity and distribution of DOX were assessed at 6, 24, and 48 h after injection. Ex vivo imaging was utilized to examine the relative distribution of DOX in the major organs and at the tumor site. After 48 h, the major organs, including the tumor, brain, lungs, heart, liver, kidneys, and spleen, were harvested and subjected to ex vivo imaging utilizing the IVIS Lumina III imaging system (PerkinElmer) to evaluate fluorescence.

#### 2.3.4 Antitumor effects and histological examination

A 100-μL aliquot of each formulation (PBS, free DOX, EV-DOX, or MBV-DOX) was administered to the mice every other day from day 0 to day 8. On day 10, a subset of tumors (n = 6) was excised for gross examination and measurement of tumor volume. The tumor volumes were calculated as previously described [34]. The body weight of the mice was measured on alternate days until they were retrieved. Additionally, organs, including the tumor, brain, lungs, heart, liver, kidneys, and spleen, were collected, and frozen sections were prepared at a thickness of 8 μm for subsequent analyses. H&E staining was routinely performed, and tumor cell apoptosis was assessed utilizing a TUNEL Apoptosis Assay Kit (C1086, Beyotime, Haimen, China) in accordance with the manufacturer's instructions. The cell nuclei were counterstained with DAPI. The percentage of apoptotic cells relative to the total cell population was quantified. DOX autofluorescence and double immunofluorescence techniques were employed to elucidate the immunophenotypes and spatial distributions of fibroblasts and macrophages. Fibroblasts and CAFs were immunostained with goat anti-vimentin antibodies (dilution 1:200, Invitrogen) and rabbit anti-FAP monoclonal antibodies (dilution 1:200, Invitrogen), respectively. The secondary antibodies used included Alexa Fluor 488-conjugated donkey anti-goat IgG and Alexa Fluor 568-conjugated goat anti-rabbit IgG (dilution 1:1000, Abcam). Macrophages of the M1 and M2 phenotypes were identified via staining with rat anti-CD86 antibodies (dilution 1:200, Invitrogen) and rabbit anti-CD163 monoclonal antibodies (dilution 1:200, Invitrogen), respectively. The secondary antibodies used included Alexa Fluor 488-conjugated goat anti-rat IgG and Alexa Fluor 568-conjugated donkey anti-rabbit IgG (dilution 1:1000, Abcam). The cell nuclei were counterstained with DAPI. The immunofluorescence staining protocol employed in this study has been previously described [34,36]. Other tumor-bearing mice (n = 8) were utilized for survival analysis. The mice were euthanized when the tumor volume exceeded 2,000 mm^3^ or when body weight loss exceeded 20 %. Kaplan–Meier survival analysis was conducted to compare the survival outcomes of A549 tumor-bearing mice across various treatment groups, and the corresponding survival curves were generated.

#### 2.3.5 Cytokine detection with enzyme-linked immunosorbent assay (ELISA)

The levels of pro-inflammatory cytokines were quantified utilizing high-sensitivity ELISA kits specific for mouse TNF-α, IL-1β, and IL-6, all procured from R&D Systems. The experimental procedures adhered strictly to the manufacturer's protocols, and quantification was conducted using a microplate reader (BioTek, Winooski, VT).

### 2.4 TMT-based quantitative proteomics analysis of acellular tumor MBVs and liquid-phase EVs

For the analysis of acellular tumor MBVs and liquid-phase EVs (n = 3), proteins were extracted utilizing an Invitrogen exosome protein extraction kit. Quantification was performed via the tandem mass tag (TMT) technique, facilitated by LC-Bio Technology Co., Ltd. (Hangzhou, China). In summary, the protein samples were subjected to enzymatic digestion with trypsin, followed by TMT labelling. After salt removal, fractionation was achieved through high-pH reversed-phase high-performance liquid chromatography (RP-HPLC). Each fraction was then quantitatively analysed via nano-LC‒MS/MS. Utilizing the raw mass spectrometry data, a database search was conducted to identify proteins, followed by an analysis of differential protein expression. A differentially expressed protein (DEP) was defined as a protein exhibiting a fold change exceeding 1.2 or below 0.83, with a p value of less than 0.05, as determined by Student's *t*-test. Principal component analysis (PCA) was employed to evaluate the differences between groups and the variability among samples. Cluster heatmaps and volcano plots were used for gene ontology (GO) enrichment analysis of the DEPs to identify biological process, cellular component, and molecular function terms associated with the DEPs, whereas Kyoto Encyclopedia of Genes and Genomes (KEGG) pathway analysis was used to explore the functions and signalling pathways of the DEPs. Protein‒protein interaction (PPI) network analysis was performed using the STRING database.

### 2.5 Statistical analyses

Statistical analyses were conducted using SPSS 25.0 (IBM Corp., Armonk, NY, USA). Student's *t*-test was used for two-group comparisons, ANOVA with Tukey's post hoc test was used for multiple group comparisons, and Kaplan–Meier analysis with the log-rank test was used for overall survival estimates. A p value less than 0.05 indicated statistical significance.

## 3 Results

### 3.1 Successful preparation and characterization of acellular tumor MBVs

**Fig. 1:**
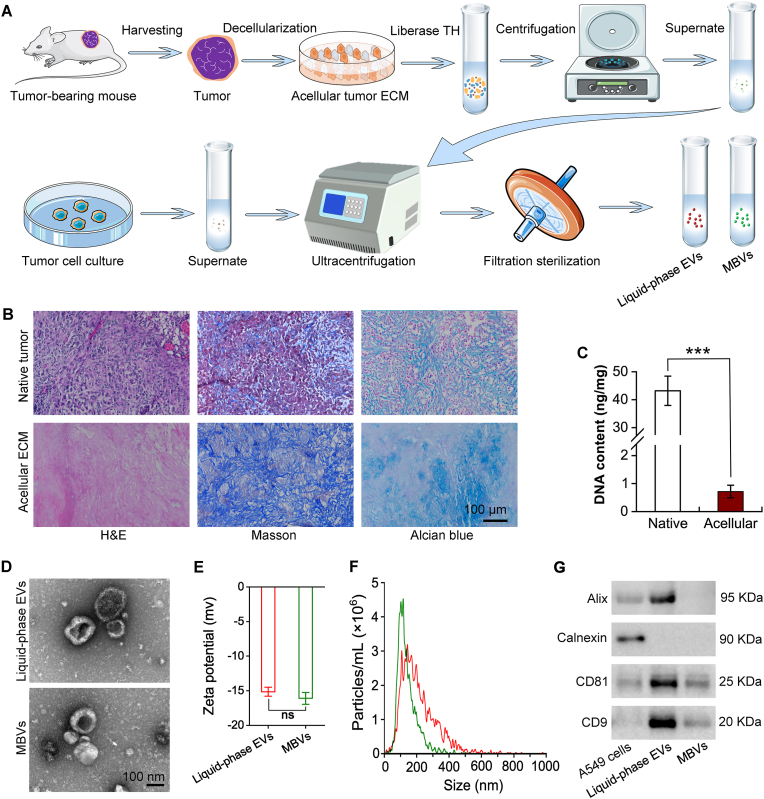
**Preparation and assessment of decellularized tumor ECM-derived MBVs.** (A) Detailed procedure for the isolation of MBVs from tumor ECM and the extraction of liquid-phase EVs from the supernatant of tumor cell cultures. (B) Representative light microscopy images of both native and decellularized tumor samples. H&E staining, Masson's trichrome staining, and Scott's Alcian blue staining were used to assess cell removal, collagen fibre integrity, and GAG content, respectively. (C) Quantitative analysis of DNA content in tumors before and after extraction (n = 8, ∗∗∗p < 0.001). (D) TEM images of acellular tumor MBVs and liquid-phase EVs. (E) Zeta potentials of liquid-phase EVs and acellular tumor MBVs. (F) NTA of the size distribution of acellular tumor MBVs and liquid-phase EVs. (G) Western blot analysis of the expression levels of CD9, CD81, Calnexin, and Alix in A549 cells, liquid-phase EVs and acellular tumor MBVs. (For interpretation of the references to color in this figure legend, the reader is referred to the Web version of this article.)

Fig. 1A shows the methodology employed for isolating MBVs from the ECM of tumors, as well as the extraction of liquid-phase EVs from the supernatant of tumor cell cultures. Fig. 1B shows tumor tissue derived from human lung adenocarcinoma A549 cells, which were implanted subcutaneously into SCID mice and subsequently subjected to a modified (Tris-Triton-PAA) version of the mixed protocol for decellularization. H&E staining confirmed the complete removal of cellular components from the tumor tissue. Compared with native tumor tissue, the decellularized tumor tissue showed minimal alterations in the levels of ECM components, such as collagens and GAGs, after Masson's trichrome and Scott's Alcian blue staining. The analysis of the DNA content, as illustrated in Fig. 1C, demonstrated that after decellularization, the DNA content was reduced to less than 2 % of that observed in the native group (*p* < 0.001, n = 8).

TEM analysis, as shown in Fig. 1D, demonstrated that the morphology of acellular tumor MBVs and tumor cell culture supernatant was comparable. Fig. 1E illustrates that the initial zeta potentials of both samples were comparable. Upon storage at 4 °C, as depicted in Fig. S3A, the zeta potentials remained stable for less than 120 h; however, they exhibited an increase at 240 h. NTA, shown in Fig. 1F, revealed a difference in size distribution between acellular tumor MBVs and liquid-phase EVs. Specifically, the acellular tumor MBVs predominantly exhibited diameters of approximately 100 nm, whereas the liquid-phase EVs were more frequently characterized by diameters of approximately 150 nm. The Western blot analysis presented in Fig. 1G revealed marked expression of the EV markers CD9, CD81, and Alix in the liquid-phase EV group. Conversely, the expression of these markers was notably diminished in the acellular tumor MBV group. Additionally, the negative control, calnexin, exhibited high expression levels in the A549 cell group, whereas its expression was absent in both the EV and MBV groups. Hemolytic assays demonstrated a minimal hemolysis rate for liquid-phase EVs and acellular tumor MBVs across various concentrations, remaining within an acceptable range of blood toxicity (Fig. S1).

### 3.2 The binding affinity of acellular tumor MBVs for tumor ECM, tumor cells, and stromal cells

**Fig. 2:**
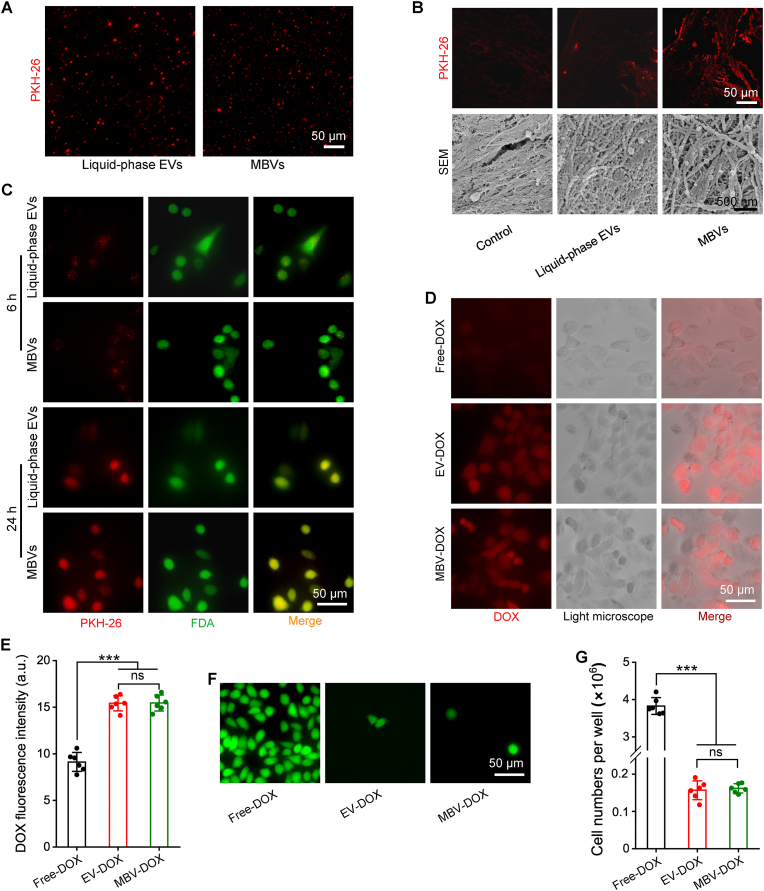
**The binding performance of liquid-phase EVs and acellular tumor MBVs with decellularized tumor ECM and A549 cells and the impact of DOX-loaded EVs on tumor cell growth.** (A) Representative images of PKH-26-labelled liquid-phase EVs and acellular tumor MBVs. (B) Representative light microscopy examination and SEM images of PKH-26-labelled liquid-phase EVs and acellular tumor MBVs incubated with decellularized tumor ECM, with decellularized tumor ECM without PKH-26 serving as a blank control. (C) Representative immunofluorescence images of PKH-26-labelled liquid-phase EVs and acellular tumor MBVs on human lung adenocarcinoma A549 cells after 6 and 24 h of incubation. (D) Images of A549 cells incubated with free DOX, EV-DOX, or MBV-DOX for 2 h. (E) DOX fluorescence intensity comparisons for the three experimental groups (n = 6). (F) Effects of free DOX, EV-DOX and MBV-DOX on the proliferation of A549 tumor cells. (G) Cell number comparisons for the three groups (n = 6). ∗∗∗p < 0.001 and ns, not significant.

PKH-26 was utilized to label acellular tumor MBVs and liquid-phase EVs. The fluorescence imaging results, as shown in Fig. 2A, demonstrated successful labelling of both types of vesicles. Fig. 2B shows the affinity of acellular tumor MBVs for decellularized tumor ECM, as evidenced by red fluorescence. Following a 6-h incubation period with PKH-26-labelled acellular tumor MBVs and control liquid-phase EVs and subsequent thorough washing, MBVs demonstrated a higher affinity for and attachment to the ECM. The fluorescence intensity of PKH-26 was most pronounced in acellular tumor MBVs, with liquid-phase extracellular vesicles exhibiting the second highest fluorescence intensity (Fig. S4A). This observation was further corroborated by scanning electron microscopy. In the control group, the surface of the decellularized tumor ECM exhibited a limited number of vesicles. Conversely, in the liquid-phase EV group, there was an observed increase in the number of vesicles on the ECM surface. Notably, the acellular tumor MBV group demonstrated the highest concentration of vesicles on the ECM surface. These findings suggest the presence of a small quantity of unseparated MBVs on the decellularized tumor ECM surface and indicate that the acellular tumor MBVs have a stronger affinity for the ECM than do liquid-phase EVs. Fluorescence imaging of the blank control revealed an absence of red staining; however, a few EVs, which were identified as MBVs, were observed on the surface of the ECM. Fig. 2C shows the experimental setup, in which PKH-26-labelled acellular tumor MBVs and liquid-phase EVs were incubated with A549 cells for 6 and 24 h. The results indicate that granular vesicles had entered the cells after 6 h, predominantly localizing near the cell membrane and gradually diffusing into the cytoplasm. By 24 h, the vesicles were no longer visible within the cells, and the PKH-26 dye had dispersed. At the 6- and 24-h time points, the PKH-26 fluorescence intensity was similar between the liquid-phase EV group and the acellular tumor MBV group (Fig. S4B). The loading efficiency of DOX in both liquid-phase EVs and acellular tumor MBVs following sonication treatment is approximately 30 %, which is significantly higher compared to the efficiency achieved through incubation, which is around 5 % (Fig. S2). DOX was released from both EV-DOX and MBV-DOX formulations in a controlled manner, with no initial burst release observed. Importantly, a significantly higher proportion of the drug was released at pH 5.5, with EV-DOX and MBV-DOX exhibiting release percentages of 61.36 ± 3.32 % and 63.75 ± 3.35 %, respectively. Conversely, at pH 7.4, the release percentages were substantially lower, recorded at 32.71 ± 1.71 % for EV-DOX and 33.31 ± 1.74 % for MBV-DOX after 24 h (Fig. S3B). The cytotoxic effects of MBVs and MBV-DOX on L929 and BEND3 cells, as illustrated in Fig. S5, indicate that both liquid-phase EVs and MBVs exhibit minimal cytotoxicity toward normal cells. Furthermore, EV-DOX and MBV-DOX demonstrate reduced cytotoxicity compared to free DOX when applied to normal cells. As shown in Fig. 2D and quantified according to fluorescence intensity in Fig. 2E, A549 cells were incubated for 2 h with free DOX, MBV-DOX, or EV-DOX. The fluorescence intensities observed in the EV-DOX and MBV-DOX groups were comparable, whereas the free DOX group presented significantly lower fluorescence intensity than both the EV-DOX and MBV-DOX groups did (*p* < 0.001 compared to both the EV-DOX and MBV-DOX groups, n = 6). Fig. 2F and G show that there was a statistically significant reduction in the number of viable cells in the EV-DOX and MBV-DOX groups (both *p* < 0.001 compared to the free DOX group, n = 6) following 72 h of treatment. These findings suggest that both DOX-loaded liquid-phase EVs and DOX-loaded acellular tumor MBVs effectively inhibited the proliferation of human A549 lung cancer cells.

**Fig. 3:**
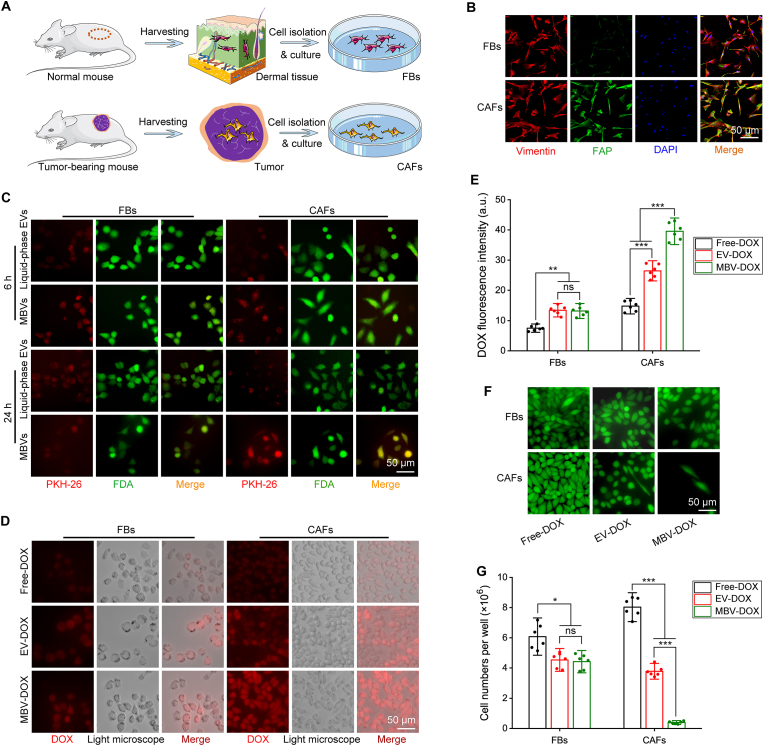
**The affinity of liquid-phase EVs and acellular tumor MBVs for fibroblasts and CAFs and the effect of DOX-loaded vesicles on cell growth. (A)** Schematic diagram of the isolation and culture of fibroblasts and CAFs. (B) Fibroblasts and CAFs were identified by dual staining with vimentin and FAP, and nuclei were stained with DAPI. (C) Representative immunofluorescence images of PKH-26-labelled liquid-phase EVs and acellular tumor MBVs interacting with fibroblasts and CAFs after 6 and 24 h of incubation. (D) Images of fibroblasts and CAFs incubated with free DOX, EV-DOX or MBV-DOX for 2 h. (E) DOX fluorescence intensity comparisons among the three groups after incubation with fibroblasts and CAFs (n = 6). (F) Effects of free DOX, EV-DOX and MBV-DOX on the proliferation of fibroblasts and CAFs. (G) Cell number comparisons among the three groups after incubation with fibroblasts and CAFs (n = 6). ∗*p* < 0.05, ∗∗*p* < 0.01, ∗∗∗*p* < 0.001 and ns, not significant.

Fig. 3A shows the methodology employed for the extraction and culture of mouse fibroblasts and CAFs from the subcutaneous tissues of mice and the tumor tissues of tumor-bearing mice, respectively. Fig. 3B shows the successful identification of fibroblasts and CAFs via vimentin and FAP immunofluorescence double staining, in which the fibroblasts presented positive staining for vimentin, whereas the CAFs presented double-positive staining for both vimentin and FAP. Fig. 3C shows the incubation of PKH-26-labelled acellular tumor MBVs and liquid-phase EVs with fibroblasts and CAFs for 6 and 24 h. At the 6-h mark, the red-stained vesicles were observed to have entered the fibroblasts, predominantly localizing near the cell membrane and beginning to diffuse into the cytoplasm. By 24 h, PKH-26 was distributed more diffusely within the cells. Compared with those in the EV group, the CAFs in the MBV group presented a greater number of red-stained vesicles that entered the cells at 6 h and further diffused at 24 h. In merged images, cells stained with both PKH-26 and the live cell stain FDA exhibit a brownish-yellow color. At the 6- and 24-h time intervals, the PKH-26 fluorescence intensity was comparable between the liquid-phase EV group and the acellular tumor MBV group in fibroblasts (Fig. S4C). However, the acellular tumor MBV group exhibited a higher fluorescence intensity in CAFs (Fig. S4D). Fig. S5 illustrates the temporal progression of PKH-26-labelled vesicle appearance in CAFs. After 2 h of incubation, a minimal presence of red-stained vesicles was observed. Between 4 and 24 h, PKH-26-labelled vesicles were progressively detected in the cell membrane, cytoplasm, and nucleus. The acellular tumor MBV group exhibited a higher abundance of vesicles and more intense fluorescence compared to the liquid-phase EV group. As shown in Fig. 3D and quantified in Fig. 3E, following a 2-h incubation of fibroblasts with free DOX, EV-DOX, or MBV-DOX, the fluorescence intensity was observed to be the weakest in the free DOX group, whereas the EV-DOX and MBV-DOX groups presented comparable fluorescence intensities. In contrast, among the CAFs treated with DOX, the CAFs in the MBV-DOX group presented the highest fluorescence intensity, whereas the CAFs in the free DOX group presented the lowest fluorescence intensity. Fig. 3F and G show that there was a marked reduction in the number of viable cells in the EV-DOX and MBV-DOX groups following 72 h of treatment. This observation suggested that both DOX-loaded liquid-phase EVs and DOX-loaded acellular tumor MBVs effectively inhibited the proliferation of fibroblasts and CAFs. Importantly, the MBV-DOX group showed the most pronounced decrease in the number of CAFs.

**Fig. 4:**
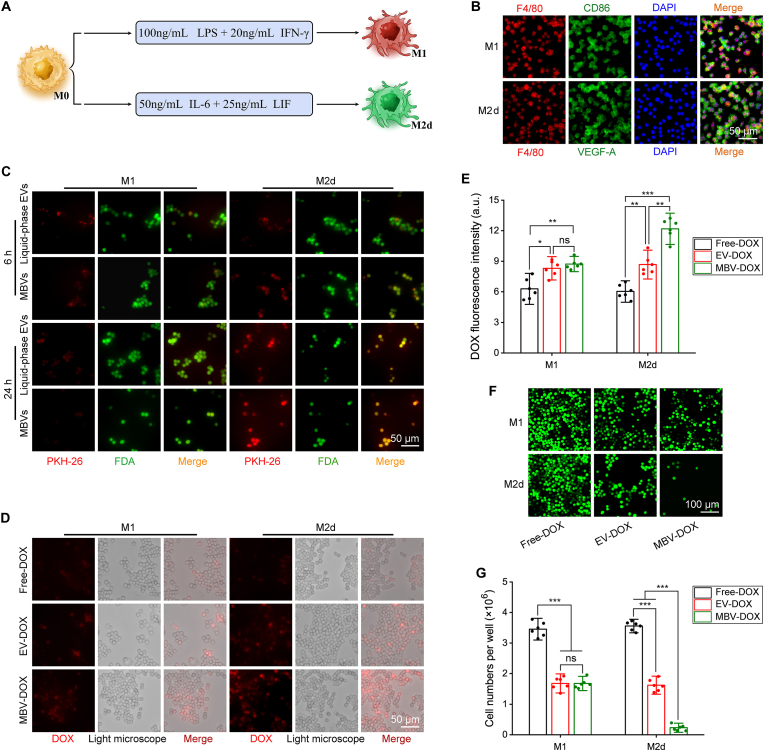
**The affinity of liquid-phase EVs and acellular tumor MBVs for M1 and M2d macrophages and the effect of DOX-loaded versions on macrophage growth.** (A) Schematic diagram showing the induction of M1 and M2d macrophage polarization from M0 macrophages. (B) M1 macrophages were characterized via dual immunostaining with the F4/80 and CD86 markers, while M2d macrophages were identified via dual staining with F4/80 and VEGF-A. Nuclear staining was performed with DAPI. (C) Representative immunofluorescence images of PKH-26-labelled liquid-phase EVs and acellular tumor MBVs interacting with M1 and M2d macrophages after 6 and 24 h of incubation. (D) Images of M1 and M2d macrophages incubated with free DOX, EV-DOX or MBV-DOX for 2 h. (E) DOX fluorescence intensity comparisons of the three groups after incubation with M1 and M2d macrophages (n = 6). (F) Effects of free DOX, EV-DOX and MBV-DOX on the proliferation of M1 and M2d macrophages. (G) Cell number comparisons among the three groups after incubation with M1 and M2d macrophages (n = 6). ∗*p* < 0.05, ∗∗*p* < 0.01, ∗∗∗*p* < 0.001 and ns, not significant.

Fig. 4A shows the methodology used to induce the polarization of M1 and M2d macrophages from M0 macrophages. Fig. 4B shows the successful identification of M1 macrophages via F4/80 and CD86 double immunofluorescence staining, while M2d macrophages were effectively identified via F4/80 and VEGF-A double immunofluorescence staining. Fig. 4C shows the incubation of PKH-26-labelled liquid-phase EVs and acellular tumor MBVs with M1 and M2d macrophages for 6 and 24 h. At 6 h post-incubation, red-stained vesicles were observed to have entered M1 macrophages, predominantly localizing near the cell membrane and subsequently diffusing into the cytoplasm. PKH-26 was found to diffuse within the cells at 24 h. In contrast, compared with those in the liquid-phase EV group, the M2d macrophages in the MBV group presented a greater number of red-stained vesicles that entered the cells at 6 h and diffused into the cytoplasm at 24 h. Within merged images, cells stained with both PKH-26 and the live cell stain FDA appeared brownish-yellow. At the 6- and 24-h time points, the PKH-26 fluorescence intensity was similar between the liquid-phase EV group and the acellular tumor MBV group in M1 macrophages (Fig. S4E). In contrast, the acellular tumor MBV group demonstrated a significantly higher fluorescence intensity in M2d macrophages (Fig. S4F).As shown in Fig. 4D and quantified in Fig. 4E, following a 2-h incubation of M1 macrophages with free DOX, EV-DOX, or MBV-DOX, the fluorescence intensity observed in the free DOX group was significantly lower than that in the EV-DOX group (*p* = 0.019, n = 6) and the MBV-DOX group (*p* = 0.002, n = 6). In contrast, the fluorescence intensities of the MBV-DOX and EV-DOX groups were comparable. Among the M2d macrophages treated with DOX, the M2d macrophages in the MBV-DOX group presented the highest fluorescence intensity (*p* = 0.005 compared to the EV-DOX group, n = 6), whereas those in the free DOX group presented the lowest fluorescence intensity (*p* = 0.006 compared to the EV-DOX group, n = 6). Fig. 4F and G show that there was a substantial reduction in the number of viable cells in the EV-DOX and MBV-DOX groups following 72 h of treatment. The M1 and M2d macrophages showed reduced viability upon treatment with DOX-loaded liquid-phase EVs or DOX-loaded MBVs. Notably, the MBV-DOX group presented the most pronounced decrease in the number of M2d macrophages.

### 3.3 Pharmacokinetics of MBV-DOX

The concentration of DOX in the plasma of SCID mice treated with MBV-DOX was measured using HPLC. The plasma concentration–time profile of DOX is depicted in Fig. S7, and the pharmacokinetic parameters are detailed in Table S1. Following an intravenous administration of 5 mg/kg DOX, the pharmacokinetic analysis revealed that the maximum plasma concentration (C_max_) for MBV-Dox and EV-Dox was 13.12 ± 0.61 μg/mL and 11.61 ± 0.57 μg/mL, respectively, occurring at 0.23 and 0.22 h post-injection. In contrast, the C_max_ for free DOX was 2.45 ± 0.13 μg/mL, observed at 0.11 h post-injection. The elimination half-life (t1/2) was determined to be 2.31, 2.14, and 0.56 h, respectively. The area under the concentration-time curve (AUC_0-t_) was 313.67 ± 41.13, 283.46 ± 35.87, and 14.25 ± 4.69 μg h/mL. The concentration of DOX decreased rapidly within 10 h for both MBV-Dox and EV-Dox, followed by a slow distribution phase and a final elimination phase. These findings suggest that EV-Dox and MBV-Dox are rapidly cleared from the bloodstream and subsequently distributed into tissues throughout the body.

### 3.4 In vivo imaging and antitumor efficacy of MBV-DOX in tumor-bearing mice

**Fig. 5:**
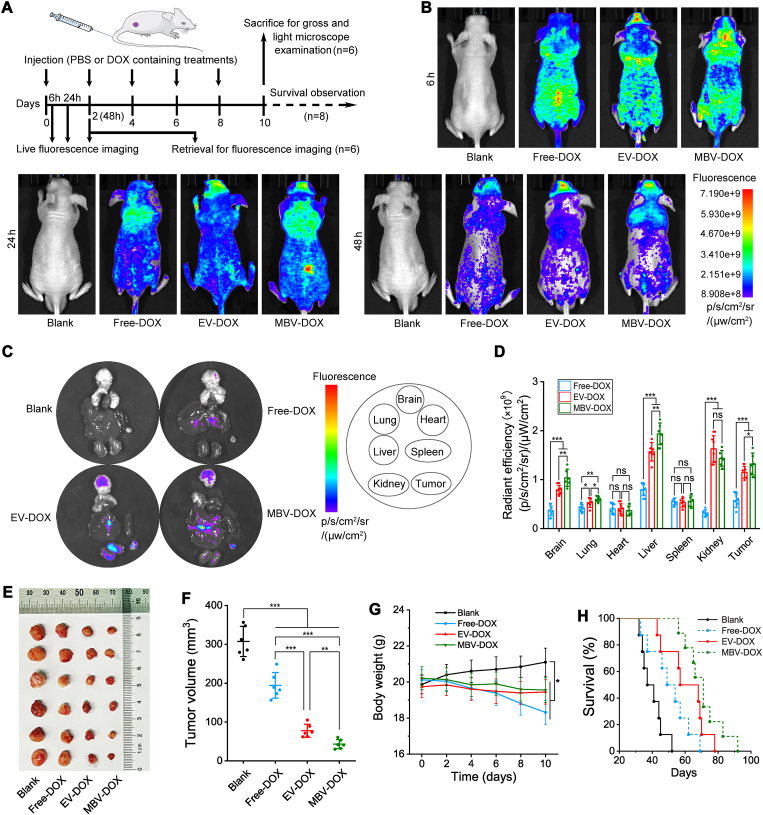
**In vivo and ex vivo fluorescence imaging and tumor progression in mice.** (A) Schematic diagram of the various treatment protocols. (B) Representative in vivo fluorescence images of tumor-bearing nude mice following the administration of PBS, free DOX, EV-DOX, or MBV-DOX. (C) Representative ex vivo fluorescence images of the brain, lungs, heart, liver, spleen, kidneys, and tumors of A549 tumor-bearing mice 48 h post-treatment in each of the four groups. (D) Quantitative comparison of fluorescence intensity across the seven organs for the free-DOX, EV-DOX, and MBV-DOX groups (n = 6). (E) Bulk gross images of tumors from each experimental group. (F) Comparative analysis of tumor volume across the four groups at the 10-day endpoint (n = 6). (G) Body weights of mice (n = 6). (H) Kaplan–Meier survival analysis of A549 tumor-bearing mice after intravenous administration of different formulations (n = 8). ∗*p* < 0.05, ∗∗*p* < 0.01, and ∗∗∗*p* < 0.001.

To examine the in vivo distribution of MBV-DOX and evaluate its tumor-targeting efficacy, we administered PBS, free DOX, EV-DOX, or MBV-DOX to tumor-bearing nude mice via tail vein injection. Fluorescence imaging was performed 6, 24, and 48 h after administration, leveraging the intrinsic autofluorescence of DOX. Fig. 5A shows a flowchart of the animal experiments. Fig. 5B shows that the PBS group did not exhibit any fluorescence signal at 6, 24, or 48 h. In contrast, free DOX, EV-DOX, and MBV-DOX successfully localized to the tumor site within 6 h. At 24 h, MBV-DOX presented the most intense fluorescence signal at the tumor site, followed by EV-DOX, which presented the second highest intensity. Both of these signals slightly decreased at 48 h. Conversely, the free DOX group presented a very weak fluorescence signal at the tumor site at both 24 and 48 h. These findings suggest that DOX encapsulated within liquid-phase EVs and acellular tumor MBVs can effectively target tumors in vivo, thereby extending the drug's residence time at the tumor site. Among the tested formulations, MBV-DOX demonstrated the best imaging capabilities. Forty-eight hours after administration, the tumors and major organs were excised from the mice for further analysis of ex vivo fluorescence signals. The data revealed that both EV-DOX and MBV-DOX accumulated primarily in the brain, liver, and kidneys. Importantly, tumor tissues from both the EV-DOX and MBV-DOX treatment groups presented strong fluorescence signals, indicating significant drug accumulation. The MBV-DOX group presented the strongest fluorescence signals in the brain, lungs, liver, and tumor tissues (Fig. 5C and D), confirming the in vivo imaging findings. Tumor volume assessments at the 10-day endpoint, as shown in Fig. 5E and F, revealed that the MBV-DOX group had the smallest tumors, followed by the EV-DOX group, whereas the PBS group presented the largest tumors. The body weights of mice did not exhibit any significant differences among the groups receiving DOX. In contrast, the PBS control group displayed the highest body weights (Fig. 5G). Additionally, Fig. 5H shows that the PBS group had the shortest OS, with the free DOX group having the second shortest OS. In contrast, the MBV-DOX group had the longest OS.

**Fig. 6:**
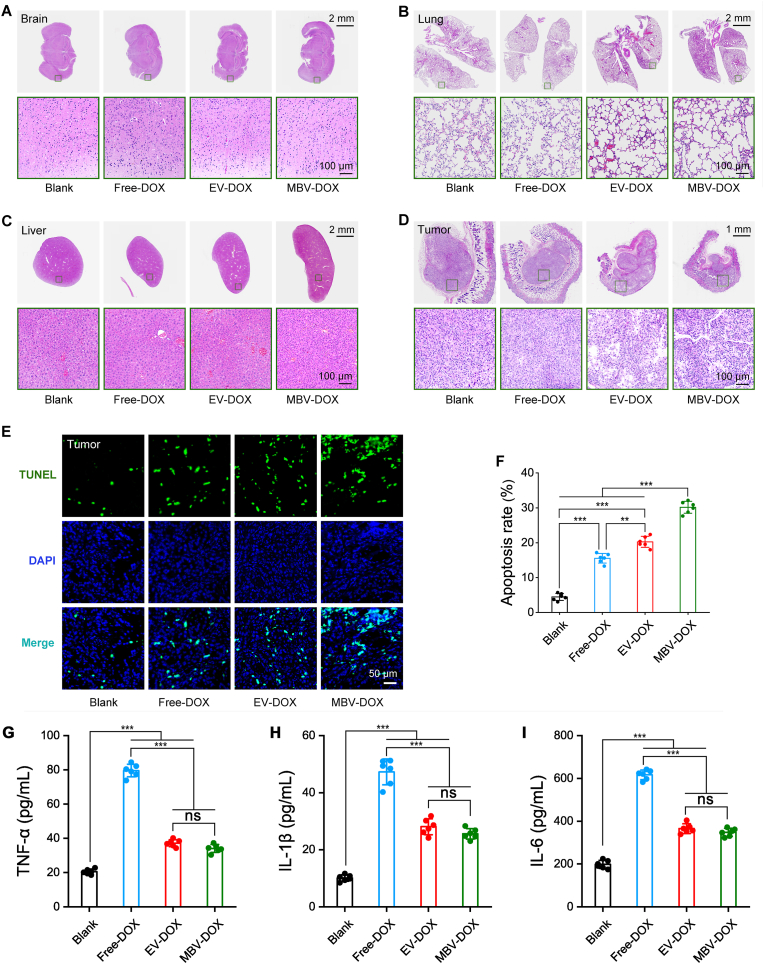
**Assessment of the tissue properties of major organs, tumor cell apoptosis and pro-inflammatory cytokine levels in tumor-bearing mice subjected to various treatments.** (A–D) H&E staining of brain (A), lung (B), liver (C), and tumor (D) tissues obtained from tumor-bearing mice from different experimental groups. (E) TUNEL assay to detect apoptosis in the four groups, with apoptotic cells visualized via TUNEL staining and nuclei counterstained with DAPI. (F) Quantification of apoptosis rates among the four groups. (G–I) Quantification of pro-inflammatory cytokines TNF-α, IL-1β, and IL-6. ∗∗*p* < 0.01, ∗∗∗*p* < 0.001 and ns, not significant.

H&E staining was conducted on sections of internal organs from experimental mice after 10 days to assess the in vivo toxicity of MBV-DOX. No significant pathological alterations were observed in the brain, lung, liver, heart, spleen, or kidney sections across all of the experimental groups (Fig. 6A–C and Fig. S8). Notably, the tumor tissues in the EV-DOX and MBV-DOX groups demonstrated increased tumor necrosis compared to that in the PBS and free DOX groups (Fig. 6D). The results of the TUNEL assay to assess apoptosis and apoptosis rate analysis indicated that the MBV-DOX group exhibited the highest level of tumor cell apoptosis, followed by the EV-DOX group. Conversely, the PBS control group presented the lowest rate of apoptosis (Fig. 6E and F). As illustrated in Fig. 6G through 6I, the group treated with free DOX exhibited the highest concentrations of TNF-α, IL-1β, and IL-6, whereas the PBS control group demonstrated the lowest levels of these cytokines. The MBV-DOX and EV-DOX groups displayed comparable levels of TNF-α, IL-1β, and IL-6.

**Fig. 7:**
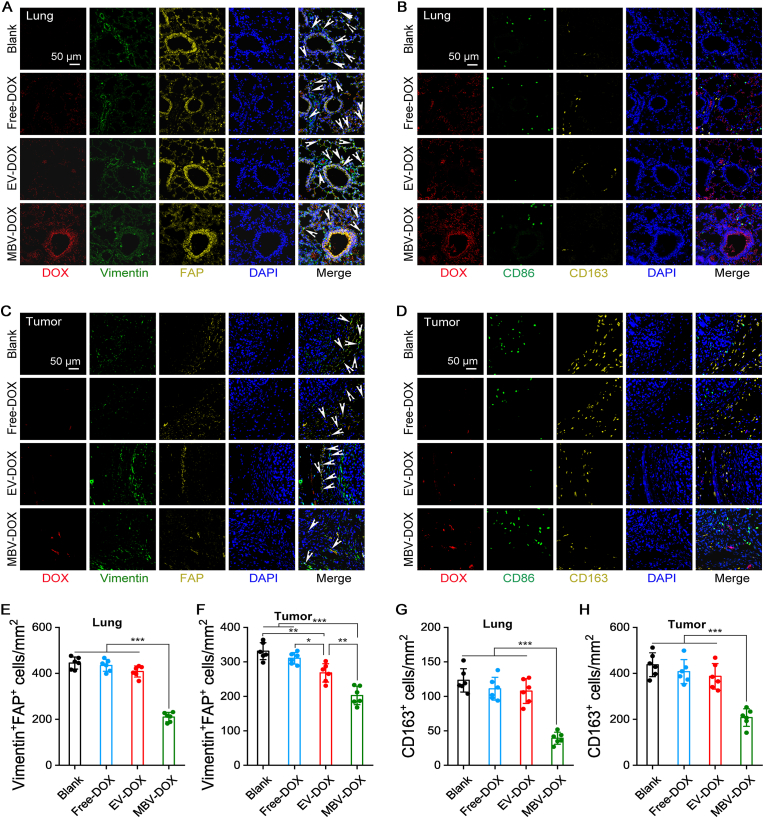
**Immunofluorescence staining to identify fibroblasts and macrophages within lung and tumor tissues.** (A) Representative immunofluorescence images of lung tissues showing vimentin-positive cells (green) and FAP-positive cells (yellow), alongside DOX autofluorescence-positive cells (red) and nuclei stained with DAPI (blue). Cancer-associated fibroblasts (CAFs) were positive for both vimentin and FAP (white arrows). (B) Representative immunofluorescence images of lung tissues showing CD86-positive cells (green, indicative of M1 macrophages) and CD163-positive cells (yellow, indicative of M2 macrophages), in addition to DOX autofluorescence-positive cells (red) and nuclei stained with DAPI (blue). (C, D) Representative fluorescence images of tumor tissues containing fibroblasts and CAFs (C, white arrows) alongside M1 and M2 macrophages (D). The cells treated with DOX exhibited red fluorescence, while the nuclei were stained blue. (E, F) Comparison of vimentin and FAP double positive cell numbers in lung and tumor tissues. (G, H) Comparison of M2-like macrophage numbers in lung and tumor tissues. The MBV-DOX group showed the lowest cell numbers. ∗*p* < 0.05, ∗∗*p* < 0.01, and ∗∗∗*p* < 0.001. (For interpretation of the references to color in this figure legend, the reader is referred to the Web version of this article.)

Fig. 7 and Fig. S9 show the results of fluorescence staining of fibroblasts and macrophages in both internal organs and tumor tissues at the 10-day end point. The PBS control group presented no detectable DOX autofluorescence (red), whereas the MBV-DOX group presented the highest level of red autofluorescence. Notably, red DOX autofluorescence was observed in erythrocytes from all the tissues in the free DOX, EV-DOX, and MBV-DOX groups. The vimentin and FAP double positive cells, as well as CD163-positive cells, which are CAFs and M2 macrophages, respectively, were colocalized with the DOX-stained regions in the MBV-DOX group. These findings suggest that MBV-DOX effectively binds to organ-resident and tumor-associated stromal cells. A reduced number of CAFs and M2 macrophages was observed in the organs and tumor of the MBV-DOX group, which was likely attributable to the cytotoxic effects of MBV-DOX on these cells. Furthermore, the tracheal epithelium exhibited FAP positivity, which also overlapped with the red autofluorescence of DOX (Fig. 7A).

### 3.5 Proteomics analysis

**Fig. 8:**
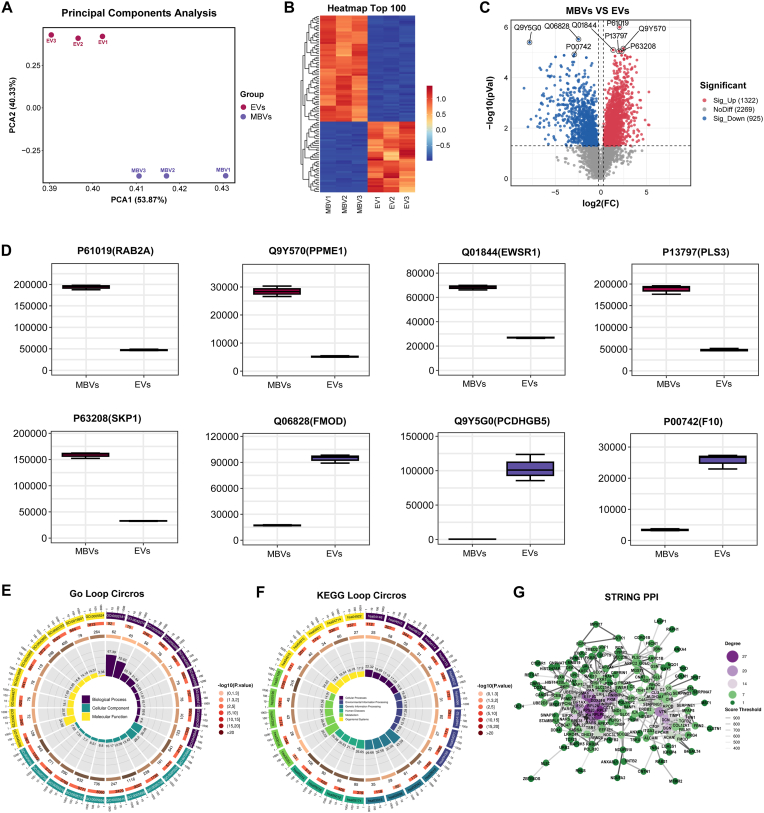
**Identification of the differentially expressed proteins between acellular tumor MBVs and liquid-phase EVs.** (A) PCA of samples to evaluate intergroup variations. (B) Heatmaps showing the top 100 differentially expressed proteins. (C) Volcano plot showing differential protein expression between the two groups. (D) Box plot displaying the expression levels of the top eight proteins with the lowest p values. (E, F) The circle diagrams for GO (E) and KEGG (F) analysis are presented. The outermost circle illustrates the GO terms or KEGG pathways that are significantly enriched, ranked by ascending P values. The outermost circle also serves as a coordinate scale indicating the number of proteins associated with each entry. Distinct colors are used to denote the classifications within the three principal GO categories or the various first-level classifications in KEGG (KEGG Level-1). The second circle illustrates the number of proteins annotated to GO entries or KEGG pathways, with the color indicating the negative logarithm (base 10) of the enrichment analysis P values. The third circle depicts the statistical distribution of differentially expressed protein counts within the GO or KEGG pathway, where the numerical value signifies the quantity. The fourth circle represents the percentage of enriched factors, denoted as the richness factor. (G) Differential analysis of PPI networks using the STRING protein interaction database. (For interpretation of the references to color in this figure legend, the reader is referred to the Web version of this article.)

PCA was performed, revealing a clear separation between the two groups, as shown in Fig. 8A. The heatmap in Fig. 8B highlights the top 100 proteins that were significantly upregulated or downregulated. A total of 4,516 proteins were identified. Among these proteins, 2,247 proteins were differentially expressed between the acellular tumor MBV group and the liquid-phase EV group. Specifically, 1,322 proteins were significantly upregulated, whereas 925 proteins were significantly downregulated, as shown in Fig. 8C. The top eight DEPs shown in Fig. 8D include five proteins—RAB2A, PPME1, EWSR1, PLS3, and SKP1—that were upregulated, and three proteins—FMOD, PCDHGB5, and F10—that were downregulated in the acellular tumor MBV group compared with the liquid-phase EV group.

We conducted GO analysis to investigate the potential functions of the DEPs. As shown in Fig. 8E and Fig. S10A, the analysis identified terms related to cellular composition, with a particular emphasis on EVs, including terms such as extracellular exosome and extracellular region. Additionally, the analysis revealed significant associations of the DEPs with RNA binding, identical protein binding, focal adhesion, and cadherin binding. To gain deeper insight into the potential biological functions of the identified DFPs, KEGG pathway analysis was performed. The identified KEGG pathways are shown in Fig. 8F, and the top 30 biological functions associated with the DEPs are presented in Fig. S10B. The analysis revealed significant enrichment of DEPs in pathways related to focal adhesion, regulation of the actin cytoskeleton, the PI3K‒Akt signalling pathway, and metabolic pathways. Among the DEPs, RPLP0, RPL4, RPS20, and EEF1G demonstrated a significant level of connectivity, engaging in interactions with numerous proteins within the PPI network (Fig. 8G).

## 4 Discussion

Nanoparticle delivery systems, which include synthetic nanoparticles, liposomes, and EVs, have been meticulously engineered to improve the pharmacokinetic and pharmacodynamic characteristics of therapeutic agents such as drugs, proteins, and nucleic acids [45,46]. Nonetheless, the clinical implementation of liposomes is impeded by several issues, including insufficient long-term stability, increased toxicity, drug leakage, and rapid clearance from the circulatory system [47]. Similarly, synthetic nanoparticles face challenges related to limited biocompatibility and potential toxicity [48]. The utilization of natural carrier systems presents a promising approach to addressing these obstacles in the delivery of therapeutic agents. Owing to their natural origin, EVs exhibit superior biocompatibility, stability, extended circulation half-life, and reduced toxicity in comparison to liposomes and synthetic polymer nanoparticles [49]. However, exosomes, which are the smallest extracellular vesicles formed through endocytosis, encounter difficulties in drug delivery for cancer treatment due to inefficient drug loading [50]. MBVs are a novel category of nanoscale vesicles that are secreted by cells and incorporated into the ECM, and they have substantial potential as platforms for the targeted delivery of gene and drug therapies [17]. MBVs have been isolated from decellularized tissues, demonstrating their capacity to remain associated with the collagen matrix post-decellularization. They are subsequently released only upon targeted enzymatic degradation, as shown in previous studies [26]. A prerequisite for this process is the ability to disrupt of the ECM structure associated with MBVs while ensuring that the MBVs remain intact. The ECM constitutes a complex three-dimensional network comprised of proteins, polysaccharides, and other macromolecules. This structure affords substantial physical and chemical protection to MBVs, safeguarding them from dissolution by detergents. Upon exposure to detergents, such as Triton X-100, the ECM initially absorbs and mitigates the detergent's impact, thereby attenuating or postponing the direct deleterious effects on the vesicle membrane. Vesicle surfaces typically feature specific proteins, such as integrins, which exhibit high-affinity binding to particular ECM components, including fibronectin and laminin. This strong interaction facilitates the sequestration of vesicles within distinct ECM microdomains, or niches. Additionally, the GAGs within the ECM, such as heparan sulphate, possess negative charges, while some vesicle membrane surfaces also bear charges. This electrostatic interaction may further modify the local microenvironment, thereby enhancing vesicle protection. Detergents are effective at dissolving films, but during decellularization, exposed vesicles on the ECM can be damaged or leak. Detergent safety is relative; ionic detergents like SDS are more aggressive than non-ionic ones like Triton X-100. Thus, Triton X-100 was selected for this study. Longer processing increases the risk of damage to the ECM and vesicles. Before enzymatic hydrolysis, using optimized, mild detergent-based methods is relatively safe and helps preserve most MBVs' integrity. ECM dissolution techniques include collagenase, releasing enzymes, protease K, and enzyme-free potassium chloride solution [35]. MBV isolation methods include ultracentrifugation, ultrafiltration, density barrier techniques, and size exclusion chromatography. Previous studies have reported that MBVs isolated via ultracentrifugation after collagenase dissociation or releasing enzyme application have high purity and significant biological activity [35]. In the present study, Liberase TH was employed to effectively degrade ECM of decellularized tumors, whereas ultracentrifugation was utilized for the extraction of tumor ECM-derived MBVs.

In this study, the presence and the physical and chemical properties of MBVs derived from decellularized tumors were systematically evaluated. The findings from NTA and TEM revealed that acellular tumor MBVs, approximately 100 nm in size and smaller than liquid-phase EVs, which measure approximately 150 nm, were successfully isolated from decellularized tumors. The size range and structural features of acellular tumor MBVs observed by the TEM in this study are consistent with those of MBVs from various tissues, as documented in previous studies [16,51,52]. The acellular tumor MBVs exhibited zeta potentials comparable to those of liquid-phase EVs. Furthermore, their zeta potentials remained stable for over 120 h, suggesting a limited stability at 4 °C. Western blot analysis further confirmed that the properties of the obtained MBVs differ from those of tumor cell-derived liquid-phase EVs. Our findings indicate the successful preparation of acellular tumor MBVs, whose characteristics are consistent with those of MBVs derived from other biomaterials.

While drugs and molecules are effective in treating tumors, their main limitation is the lack of a targeted drug delivery system, which can result in potential side effects during clinical treatment [53]. Tumor cell-derived EVs have been shown to localize to specific recipient cells on the basis of the proteins expressed on their surfaces. Various therapeutic agents can be encapsulated within tumor cell-derived EVs from different cell lines to achieve targeted delivery. By employing these engineering strategies, therapeutic cargo can be loaded more efficiently and directed towards specific tissues [54]. EVs derived from tumor cells demonstrate enhanced targeting of and uptake efficiency by the parent tumor. Consequently, these tumor cell-derived EVs can be engineered to incorporate therapeutic agents while minimizing pathogenic elements, thus offering a personalized EV-based therapeutic approach that targets both the primary tumor and its metastatic sites [[55], [56], [57]]. An optimal anticancer drug delivery system, when administered systemically, should not only achieve efficient cellular uptake and exert significant cytotoxic effects on cancer cells and tumor-associated stromal cells but also exhibit effective tumor accumulation and penetration [58]. This ensures effective targeting of both cancer cells and the surrounding stroma. Since tumor cell-derived EVs cannot specifically target the tumor ECM and stromal cells, this study investigated the in vitro affinity of MBV-DOX for tumors and tumor stroma, as well as its in vivo and ex vivo biodistribution. The uptake of DOX by fibroblasts CAFs was analysed following incubation with free DOX, EV-DOX, or MBV-DOX. The fluorescence intensity measurements indicated that the free DOX group exhibited the lowest fluorescence intensity among the fibroblasts, whereas the EV-DOX and MBV-DOX groups demonstrated comparable fluorescence intensities. Conversely, within the CAFs treated with DOX, the MBV-DOX group exhibited the highest fluorescence intensity, while the free DOX group showed the lowest. This suggests that the MBV-DOX group achieved the highest DOX uptake by CAFs. The primary mechanism for EV uptake is endocytosis, during which some internalized EVs undergo fusion with endosomes and/or lysosomes, as described by Joshi et al. [59]. This fusion process facilitates the gradual release of EV cargo into the cytosol. This mechanism may explain the observed reduction in vesicle visibility and the dispersion of PKH-26 fluorescence at 24 h. Acellular tumor MBVs mainly localized to parent tumor cells, CAFs, and TAMs, suggesting that they primarily originate from tumor and stromal cells, especially CAFs. Thus, using these MBVs to deliver therapeutic agents to these cells is a promising strategy.

Premetastatic niches facilitate cancer cell spread before organ-specific metastases develop, adversely affecting the outcome of surgical treatment [60]. The ECM, tumor cells, CAFs, and TAMs play crucial roles in the development of these niches [[61], [62], [63]]. Activated fibroblasts within tumors, known as CAFs, are crucial in tumor biology, particularly in terms of collagen deposition and immunosuppression, and they have been a focus of substantial clinical and preclinical research [64]. TAMs often present an immunosuppressive M2-like phenotype that is linked to poor cancer outcomes [65,66]. Emerging research indicates that reprogramming TAMs may be a promising therapeutic approach for cancer [[67], [68], [69]]. The ECM acts as a "soil" for tumor growth, presenting a new target for antitumor treatments. EVs contain diverse proteins and lipids, some of which interact with the ECM via covalent or hydrogen bonds [70]. This study revealed that acellular tumor MBVs strongly bind to the tumor ECM, aiding in locating both primary and metastatic tumors. In vitro tests indicate that these MBVs have a strong affinity for the tumor ECM, tumor cells, CAFs, and TAMs, whereas liquid-phase EVs mainly target tumor cells. DOX-loaded acellular tumor MBVs have cytotoxic and therapeutic effects on both parent tumor cells and tumor-associated stromal cells, whereas DOX-loaded liquid-phase EVs affect only parent tumor cells. Additionally, DOX-loaded acellular tumor MBVs target and kill CAFs and TAMs in metastasis-prone organs such as the brain, lungs, and liver. Taken together, the results of this study demonstrated that acellular tumor MBVs can bind to the tumor ECM, parental tumor cells, and stromal cells. This specificity facilitates precise tracking and treatment of tumors from both cellular and stromal perspectives when loaded with imaging and therapeutic agents, thereby establishing a robust foundation for future clinical translation. MBVs can be isolated from fresh tumor tissue obtained through clinical surgical procedures and inherently possess homing capabilities towards both tumor cells and the tumor stroma, including ECM and stromal cells. Furthermore, once modified, tumor-derived MBVs can be utilized for imaging tumors and their stroma, as well as for treating tumor cells and their microenvironment. This capability holds significant promise for effectively monitoring and addressing postoperative tumor recurrence and metastasis.

Characterizing MBV cargoes is crucial for understanding their roles in matrix homeostasis and cell signalling. Like other EVs, MBVs are rich in proteins, miRNAs, and lipids, which are vital for ECM functions such as cell signalling and inflammation regulation [18,71]. Given the tissue-specific cargoes of MBVs and their influence on the biological activity of the parent matrix, it is crucial to analyse their cargo proteins to understand their cellular signalling potential. Mass spectrometry is a promising technique for analysing the protein composition of MBVs, with the potential for accurately identifying their protein cargoes and cell surface receptors, and it has been used successfully to characterize exosomes [19,72]. The results obtained from TMT labelling followed by mass spectrometry-based proteomic analyses highlight the crucial role of MBVs in the transport of bioactive proteins. A comparative analysis of protein composition between acellular tumor MBVs and liquid-phase EVs revealed significant differences. In particular, there is a notably higher abundance of RAB2A, a membrane-associated protein involved in vesicular fusion and trafficking [73], in acellular tumor MBVs. In this study, GO enrichment analysis identified terms related to the cellular composition, with a particular emphasis on EVs, such as extracellular exosome and extracellular region. Additionally, the analysis revealed significant associations with terms related to RNA binding, identical protein binding, focal adhesion, and cadherin binding, which have been implicated in the homing of proteins to parent cells and subsequent binding affinity. KEGG pathway analysis revealed significant enrichment of DEPs related to focal adhesion, actin cytoskeleton regulation, PI3K-Akt signalling, and metabolic pathways. We subsequently constructed a PPI network of the DEPs using the STRING database. Our analysis identified four biological hub genes with a degree greater than 20: RPLP0, RPL4, RPS20, and EEF1G. These hub genes are likely to play pivotal roles in the regulation of tumorigenesis and protein targeting processes [74]. In summary, proteomic analysis revealed that the significant DEPs between acellular tumor MBVs and liquid-phase EVs are associated with protein binding and adhesion. This relationship may underlie the affinity of acellular tumor MBVs for the ECM, tumors, and tumor-associated stromal cells.

This study has several limitations. First, the tumors used were generated from human tumor cells implanted in mice, resulting in acellular tumor MBVs derived from both human tumor cells and mouse stromal cells. Consequently, these MBVs may not accurately represent those derived solely from the human tumor ECM. Second, the current investigation focused on acellular tumor MBVs originating from lung cancer cells, leaving the characteristics of MBVs from other cancer types unexplored. Finally, this study involved a comparative analysis of DEPs between acellular tumor MBVs and liquid-phase EVs. Additional components of MBVs, including lipids and miRNAs, may contribute to their affinity for the ECM, tumor cells, and stromal cells. Future research should focus on investigating acellular tumor MBVs derived from various types of human solid tumors. Future studies should perform comprehensive lipidomic analyses and RNA sequencing of acellular tumor MBVs and liquid-phase EVs.

## 5 Conclusions

In conclusion, MBVs were successfully isolated from the ECM of decellularized tumors. Compared with liquid-phase EVs, acellular tumor MBVs had smaller dimensions. They carried numerous bioactive molecules, including proteins that interact with the ECM, tumor cells, and stromal cells. Furthermore, acellular tumor MBVs were shown to possess specific chemotactic properties both in vitro and in vivo. These findings offer valuable insights for future investigations into the role of tumor ECM-derived MBVs in targeted tumor therapy and the regulation of premetastatic niches.

## CRediT authorship contribution statement

**Zheng-Hong Chen:** Writing – original draft, Project administration, Investigation. **Ye-Rong Hu:** Resources, Data curation. **Xing-Bo Yue:** Software. **Kun Zhao:** Supervision. **Huan Yang:** Validation. **Zhi-Gang Liu:** Visualization. **Rui Xu:** Validation, Formal analysis. **Wei-Dong Lü:** Writing – review & editing, Funding acquisition, Conceptualization.

## Declaration of competing interest

The authors declare that they have no known competing financial interests or personal relationships that could have appeared to influence the work reported in this paper.

## Data Availability

Data will be made available on request.

## References

[1] Flugel C.L., Majzner R.G., Krenciute G., Dotti G., Riddell S.R., Wagner D.L., Abou-El-Enein M. (2023). Overcoming on-target, off-tumour toxicity of CAR T cell therapy for solid tumours. Nat. Rev. Clin. Oncol..

[2] de Visser K.E., Joyce J.A. (2023). The evolving tumor microenvironment: from cancer initiation to metastatic outgrowth. Cancer Cell.

[3] Nishida-Aoki N., Ochiya T. (2024). Impacts of tissue context on extracellular vesicles-mediated cancer-host cell communications. Cancer Sci..

[4] Mao X., Wu S., Huang D., Li C. (2024). Complications and comorbidities associated with antineoplastic chemotherapy: rethinking drug design and delivery for anticancer therapy. Acta Pharm. Sin. B.

[5] Ding X., Wang W., Wang Y., Bao X., Wang Y., Wang C., Chen J., Zhang F., Zhou J. (2014). Versatile reticular polyethylenimine derivative-mediated targeted drug and gene codelivery for tumor therapy. Mol. Pharm..

[6] Dixson A.C., Dawson T.R., Di Vizio D., Weaver A.M. (2023). Context-specific regulation of extracellular vesicle biogenesis and cargo selection. Nat. Rev. Mol. Cell Biol..

[7] Walker S., Busatto S., Pham A., Tian M., Suh A., Carson K., Quintero A., Lafrence M., Malik H., Santana M.X., Wolfram J. (2019). Extracellular vesicle-based drug delivery systems for cancer treatment. Theranostics.

[8] Yong T., Zhang X., Bie N., Zhang H., Zhang X., Li F., Hakeem A., Hu J., Gan L., Santos H.A., Yang X. (2019). Tumor exosome-based nanoparticles are efficient drug carriers for chemotherapy. Nat. Commun..

[9] Li Y., Wang Y., Zhang Y., Zhu Y., Dong Y., Cheng H., Zhang Y., Wang Y., Li Z., Gao J. (2024). Engineered mesenchymal stem cell-derived extracellular vesicles: kill tumors and protect organs. Theranostics.

[10] Hadad S., Khalaji A., Sarmadian A.J., Sarmadian P.J., Janagard E.M., Baradaran B. (2024). Tumor-associated macrophages derived exosomes; from pathogenesis to therapeutic opportunities. Int. Immunopharmacol..

[11] Duan X., Yu X., Gan J. (2024). Extracellular vesicle-packaged miR-4253 secreted by cancer-associated fibroblasts facilitates cell proliferation in gastric cancer by inducing macrophage M2 polarization. Cancer Biol. Ther..

[12] Santi A., Kay E.J., Neilson L.J., McGarry L., Lilla S., Mullin M., Paul N.R., Fercoq F., Koulouras G., Rodriguez Blanco G., Athineos D., Mason S., Hughes M., Thomson G., Kieffer Y., Nixon C., Blyth K., Mechta-Grigoriou F., Carlin L.M., Zanivan S. (2024). Cancer-associated fibroblasts produce matrix-bound vesicles that influence endothelial cell function. Sci. Signal..

[13] Fu Q.Y., Xiong X.P., Xia H.F., Liu X.C., Yu Z.L., Liu K.W., Zeng J., Sun Y.F., Jia J., Chen G. (2024). Spatiotemporal characteristics of tissue derived small extracellular vesicles is associated with tumor relapse and anti-PD-1 response. Cancer Lett..

[14] Li W., Zhu J., Li J., Jiang Y., Sun J., Xu Y., Pan H., Zhou Y., Zhu J. (2024). Research advances of tissue-derived extracellular vesicles in cancers. J. Cancer Res. Clin. Oncol..

[15] Mishra A., Singh P., Qayoom I., Prasad A., Kumar A. (2021). Current strategies in tailoring methods for engineered exosomes and future avenues in biomedical applications. J. Mater. Chem. B.

[16] Huleihel L., Hussey G.S., Naranjo J.D., Zhang L., Dziki J.L., Turner N.J., Stolz D.B., Badylak S.F. (2016). Matrix-bound nanovesicles within ECM bioscaffolds. Sci. Adv..

[17] Piening L.M., Wachs R.A. (2023). Matrix-bound nanovesicles: what are they and what do they do?. Cells Tissues Organs.

[18] Hussey G.S., Pineda Molina C., Cramer M.C., Tyurina Y.Y., Tyurin V.A., Lee Y.C., El-Mossier S.O., Murdock M.H., Timashev P.S., Kagan V.E., Badylak S.F. (2020). Lipidomics and RNA sequencing reveal a novel subpopulation of nanovesicle within extracellular matrix biomaterials. Sci. Adv..

[19] Peshkova M., Korneev A., Revokatova D., Smirnova O., Klyucherev T., Shender V., Arapidi G., Kosheleva N., Timashev P. (2024). Four sides to the story: a proteomic comparison of liquid-phase and matrix-bound extracellular vesicles in 2D and 3D cell cultures. Proteomics.

[20] Zeng T., Yuan P., Liang L., Zhang X., Zhang H., Wu W. (2022). Cartilaginous extracellular matrix enriched with human gingival mesenchymal stem cells derived "Matrix Bound Extracellular Vesicles" enabled functional reconstruction of tracheal defect. Adv. Sci. (Weinh.).

[21] Lazar S., Mor S., Chen J., Hao D., Wang A. (2021). Bioengineered extracellular vesicle-loaded bioscaffolds for therapeutic applications in regenerative medicine. Extracell Vesicles Circ Nucl Acids.

[22] Jo S.H., Kim C., Park S.H. (2021). Novel marine organism-derived extracellular vesicles for control of anti-inflammation. Tissue Eng Regen Med.

[23] Cramer M., Pineda Molina C., Hussey G., Turnquist H.R., Badylak S.F. (2022). Transcriptomic regulation of macrophages by matrix-bound nanovesicle-associated Interleukin-33. Tissue Eng Part A.

[24] Liu C., Chen X., Liu Y., Sun L., Yu Z., Ren Y., Zeng C., Li Y. (2023). Engineering extracellular matrix-bound nanovesicles secreted by three-dimensional human mesenchymal stem cells. Adv. Healthcare Mater..

[25] Yang J., Bahcecioglu G., Ronan G., Zorlutuna P. (2024). Aged breast matrix bound vesicles promote breast cancer invasiveness. Biomaterials.

[26] Crum R.J., Capella-Monsonis H., Chang J., Dewey M.J., Kolich B.D., Hall K.T., El-Mossier S.O., Nascari D.G., Hussey G.S., Badylak S.F. (2023). Biocompatibility and biodistribution of matrix-bound nanovesicles in vitro and in vivo. Acta Biomater..

[27] Garcia-Gareta E., Perez M.A., Garcia-Aznar J.M. (2022). Decellularization of tumours: a new frontier in tissue engineering. J. Tissue Eng..

[28] Cruz-Acuna R., Vunjak-Novakovic G., Burdick J.A., Rustgi A.K. (2021). Emerging technologies provide insights on cancer extracellular matrix biology and therapeutics. iScience.

[29] Zhang X., Chen X., Hong H., Hu R., Liu J., Liu C. (2022). Decellularized extracellular matrix scaffolds: recent trends and emerging strategies in tissue engineering. Bioact. Mater..

[30] Lv Y., Wang H., Li G., Zhao B. (2021). Three-dimensional decellularized tumor extracellular matrices with different stiffness as bioengineered tumor scaffolds. Bioact. Mater..

[31] Gentilin E., D'Angelo E., Agostini M., Astolfi L. (2022). Decellularized normal and cancer tissues as tools for cancer research. Cancer Gene Ther..

[32] DiPersio C.M., Van De Water L. (2019). Integrin regulation of CAF differentiation and function. Cancers (Basel).

[33] Lu W.D., Zhang L., Wu C.L., Liu Z.G., Lei G.Y., Liu J., Gao W., Hu Y.R. (2014). Development of an acellular tumor extracellular matrix as a three-dimensional scaffold for tumor engineering. PLoS One.

[34] Lu W.D., Sun R.F., Hu Y.R., Lu J.R., Gu L., Liu Z.G., Lei G.Y., Qiang Z., Cai L. (2018). Photooxidatively crosslinked acellular tumor extracellular matrices as potential tumor engineering scaffolds. Acta Biomater..

[35] Quijano L.M., Naranjo J.D., El-Mossier S.O., Turner N.J., Pineda Molina C., Bartolacci J., Zhang L., White L., Li H., Badylak S.F. (2020). Matrix-bound nanovesicles: the effects of isolation method upon yield, purity, and function. Tissue Eng. C Methods.

[36] Lu W.D., Liu Y.Z., Yang Y.Q., Liu Z.G., Zhao K., Lu J.R., Lei G.Y., Wang Y.Y., Cai L., Sun R.F. (2022). Effect of naturally derived surgical hemostatic materials on the proliferation of A549 human lung adenocarcinoma cells. Mater. Today Bio.

[37] Zhang Y., Chen Z.H., Zhao K., Mu Y.D., Li K.L., Yuan Z.M., Liu Z.G., Han L., Lu W.D. (2024). Acellular embryoid body and hydroxybutyl chitosan composite hydrogels promote M2 macrophage polarization and accelerate diabetic cutaneous wound healing. Mater. Today Bio.

[38] Lu W.D., Liu Y.Z., Liu Z.G., Wu C.L., Lei G.Y., Zhang X., Gao W., Hu Y.R. (2016). Effect of lyophilization technique and gamma-ray sterilization on structural, mechanical and biological properties of acellular tumor extracellular matrix scaffolds. J Biomater Tiss Eng.

[39] Yang S., Pham L.K., Liao C.P., Frenkel B., Reddi A.H., Roy-Burman P. (2008). A novel bone morphogenetic protein signaling in heterotypic cell interactions in prostate cancer. Cancer Res..

[40] Fitzgerald A.A., Weiner L.M. (2020). The role of fibroblast activation protein in health and malignancy. Cancer Metastasis Rev..

[41] Simkova A., Busek P., Sedo A., Konvalinka J. (2020). Molecular recognition of fibroblast activation protein for diagnostic and therapeutic applications. Biochim. Biophys. Acta Proteins Proteom..

[42] Schmieder A., Michel J., Schonhaar K., Goerdt S., Schledzewski K. (2012). Differentiation and gene expression profile of tumor-associated macrophages. Semin. Cancer Biol..

[43] Duluc D., Delneste Y., Tan F., Moles M.P., Grimaud L., Lenoir J., Preisser L., Anegon I., Catala L., Ifrah N., Descamps P., Gamelin E., Gascan H., Hebbar M., Jeannin P. (2007). Tumor-associated leukemia inhibitory factor and IL-6 skew monocyte differentiation into tumor-associated macrophage-like cells. Blood.

[44] Anders C.B., Lawton T.M.W., Smith H.L., Garret J., Doucette M.M., Ammons M.C.B. (2022). Use of integrated metabolomics, transcriptomics, and signal protein profile to characterize the effector function and associated metabotype of polarized macrophage phenotypes. J. Leukoc. Biol..

[45] Cao Y., Tang L., Fu C., Yin Y., Liu H., Feng J., Gao J., Shu W., Li Z., Zhu Y., Wang W. (2024). Black phosphorus quantum dot loaded bioinspired nanoplatform synergized with aPD-L1 for multimode cancer immunotherapy. Nano Lett..

[46] Tang L., Xie M., Li J., Mei Y., Cao Y., Xiao Q., Dong H., Zhang Y., Wang W. (2023). Leveraging nano-engineered mesenchymal stem cells for intramedullary spinal cord tumor treatment. Chin. Chem. Lett..

[47] Egwu C.O., Aloke C., Onwe K.T., Umoke C.I., Nwafor J., Eyo R.A., Chukwu J.A., Ufebe G.O., Ladokun J., Audu D.T., Agwu A.O., Obasi D.C., Okoro C.O. (2024). Nanomaterials in drug delivery: strengths and opportunities in medicine. Molecules (Basel, Switzerland).

[48] Sharma S., Parveen R., Chatterji B.P. (2021). Toxicology of nanoparticles in drug delivery. Current pathobiology reports.

[49] Su X., Wang H., Li Q., Chen Z. (2025). Extracellular vesicles: a review of their therapeutic potentials, sources, biodistribution, and administration routes. Int. J. Nanomed..

[50] Safaei M., Rajabi S.S., Tirgar M., Namdar N., Dalfardi M., Mohammadifar F., Goodarzi A., Farmani A.R., Ramezani V., Abpeikar Z. (2025). Exosome-based approaches in cancer along with unlocking new insights into regeneration of cancer-prone tissues. Regenerative therapy.

[51] Di Francesco D., Di Varsavia C., Casarella S., Donetti E., Manfredi M., Mantovani D., Boccafoschi F. (2024). Characterisation of matrix-bound nanovesicles (MBVs) isolated from decellularised bovine pericardium: new frontiers in regenerative medicine. Int. J. Mol. Sci..

[52] Kobayashi M., Negishi J., Ishida N., Hashimoto Y., Sasaki Y., Akiyoshi K., Kimura T., Kishida A. (2024). Effects of the matrix-bounded nanovesicles of high-hydrostatic pressure decellularized tissues on neural regeneration. Sci. Technol. Adv. Mater..

[53] Chitti S.V., Nedeva C., Manickam R., Fonseka P., Mathivanan S. (2022). Extracellular vesicles as drug targets and delivery vehicles for cancer therapy. Pharmaceutics.

[54] Kalluri R., McAndrews K.M. (2023). The role of extracellular vesicles in cancer. Cell.

[55] Zhou X., Miao Y., Wang Y., He S., Guo L., Mao J., Chen M., Yang Y., Zhang X., Gan Y. (2022). Tumour-derived extracellular vesicle membrane hybrid lipid nanovesicles enhance siRNA delivery by tumour-homing and intracellular freeway transportation. J. Extracell. Vesicles.

[56] Ge K., Ren Y., Hong Z., Mao Z., Yao B., Ye K., Jia C. (2024). Microchip based isolation and drug delivery of patient-derived extracellular vesicles against their homologous tumor. Adv. Healthcare Mater..

[57] Peng Z., Zhao T., Gao P., Zhang G., Wu X., Tian H., Qu M., Tan X., Zhang Y., Zhao X., Qi X. (2024). Tumor-derived extracellular vesicles enable tumor tropism chemo-genetherapy for local immune activation in triple-negative breast cancer. ACS Nano.

[58] Prange C.J., Hu X., Tang L. (2023). Smart chemistry for traceless release of anticancer therapeutics. Biomaterials.

[59] Joshi B.S., de Beer M.A., Giepmans B.N.G., Zuhorn I.S. (2020). Endocytosis of extracellular vesicles and release of their cargo from endosomes. ACS Nano.

[60] Wang Y., Jia J., Wang F., Fang Y., Yang Y., Zhou Q., Yuan W., Gu X., Hu J., Yang S. (2024). Pre-metastatic niche: formation, characteristics and therapeutic implication. Signal Transduct. Targeted Ther..

[61] Kong J., Tian H., Zhang F., Zhang Z., Li J., Liu X., Li X., Liu J., Li X., Jin D., Yang X., Sun B., Guo T., Luo Y., Lu Y., Lin B., Liu T. (2019). Extracellular vesicles of carcinoma-associated fibroblasts creates a pre-metastatic niche in the lung through activating fibroblasts. Mol. Cancer.

[62] Wang S.E. (2022). Extracellular vesicles in cancer therapy. Semin. Cancer Biol..

[63] Nasrollahzadeh E., Razi S., Keshavarz-Fathi M., Mazzone M., Rezaei N. (2020). Pro-tumorigenic functions of macrophages at the primary, invasive and metastatic tumor site. Cancer Immunol. Immunother..

[64] Caligiuri G., Tuveson D.A. (2023). Activated fibroblasts in cancer: perspectives and challenges. Cancer Cell.

[65] Cassetta L., Pollard J.W. (2023). A timeline of tumour-associated macrophage biology. Nat. Rev. Cancer.

[66] Li M., Lu L., Xiao Q., Maalim A.A., Nie B., Liu Y., Kahlert U.D., Shu K., Lei T., Zhu M. (2024). Bioengineer mesenchymal stem cell for treatment of glioma by IL-12 mediated microenvironment reprogramming and nCD47-SLAMF7 mediated phagocytosis regulation of macrophages. Exploration (Beijing, China).

[67] Zhang X., Li S., Malik I., Do M.H., Ji L., Chou C., Shi W., Capistrano K.J., Zhang J., Hsu T.W., Nixon B.G., Xu K., Wang X., Ballabio A., Schmidt L.S., Linehan W.M., Li M.O. (2023). Reprogramming tumour-associated macrophages to outcompete cancer cells. Nature.

[68] Chen Z., Yang S., Zhao Z., Feng L., Sheng J., Deng R., Wang B., He Y., Luo D., Chen M., Chen L., Chang K. (2024). Smart tumor cell-derived DNA nano-tree assembly for On-Demand macrophages reprogramming. Adv. Sci. (Weinh.).

[69] Li J., Yang J., Jiang S., Tian Y., Zhang Y., Xu L., Hu B., Shi H., Li Z., Ran G., Huang Y., Ruan S. (2024). Targeted reprogramming of tumor-associated macrophages for overcoming glioblastoma resistance to chemotherapy and immunotherapy. Biomaterials.

[70] Debnath K., Heras K.L., Rivera A., Lenzini S., Shin J.W. (2023). Extracellular vesicle-matrix interactions. Nat. Rev. Mater..

[71] Turner N.J., Quijano L.M., Hussey G.S., Jiang P., Badylak S.F. (2022). Matrix bound nanovesicles have tissue-specific characteristics that suggest a regulatory role. Tissue Eng Part A.

[72] Atay S., Gercel-Taylor C., Kesimer M., Taylor D.D. (2011). Morphologic and proteomic characterization of exosomes released by cultured extravillous trophoblast cells. Exp. Cell Res..

[73] Kajiho H., Kajiho Y., Scita G. (2018). Harnessing membrane trafficking to promote cancer spreading and invasion: the case of RAB2A. Small GTPases.

[74] Zhu Z., He A., Lin L., Xu C., Cai T., Lin J. (2020). Biological functions and prognostic value of RNA binding proteins in clear cell renal cell carcinoma. J. Cancer.

