# Supplementary material for: Matrix-bound nanovesicles isolated from decellularized tumors as platforms for targeting parent tumor cells and tumor-associated stromal cells
Source: Mater Today Bio. 2025 Sep 24;35:102355. doi: 10.1016/j.mtbio.2025.102355 (PMC12513100; doi:10.1016/j.mtbio.2025.102355)
Supplement: Multimedia component 1 [file mmc1.docx]

**Supporting Information**

**Matrix-bound nanovesicles isolated from decellularized tumors as platforms for targeting parent tumor cells and tumor-associated stromal cells**

Zheng-Hong Chen^a1^, Ye-Rong Hu^b1^, Xing-Bo Yue^c, d^, Kun Zhao^c^, Huan Yang^c^, Zhi-Gang Liu^c^, Rui Xu^e^, Wei-Dong Lü^c*^

*^a^Department of Integrated Chinese and Western Medicine, Tumor Hospital of Shaanxi Province, Affiliated to the Medical College of Xi'an Jiaotong University, Xi’an, Shaanxi, 710061, China*

*^b^Department of Cardiovascular Surgery, The Second Xiangya Hospital of Central South University, Changsha, Hunan, 410000, China*

*^c^Department of Thoracic Surgery, Tumor Hospital of Shaanxi Province, Affiliated to the Medical College of Xi'an Jiaotong University, Xi’an, Shaanxi, 710061, China*

*^d^Department of Surgery, The Second Affiliated Hospital of Shaanxi University of Chinese Medicine, Xianyang, Shaanxi, 712000, China*

*^e^Department of Oncology, Tumor Hospital of Shaanxi Province, Affiliated to the Medical College of Xi'an Jiaotong University, Xi’an, Shaanxi, 710061, China*

**Corresponding author, E-mail address:* [*wdlu76@aliyun.com*](mailto:wdlu76@aliyun.com)

^1^ These authors contributed equally to this work.


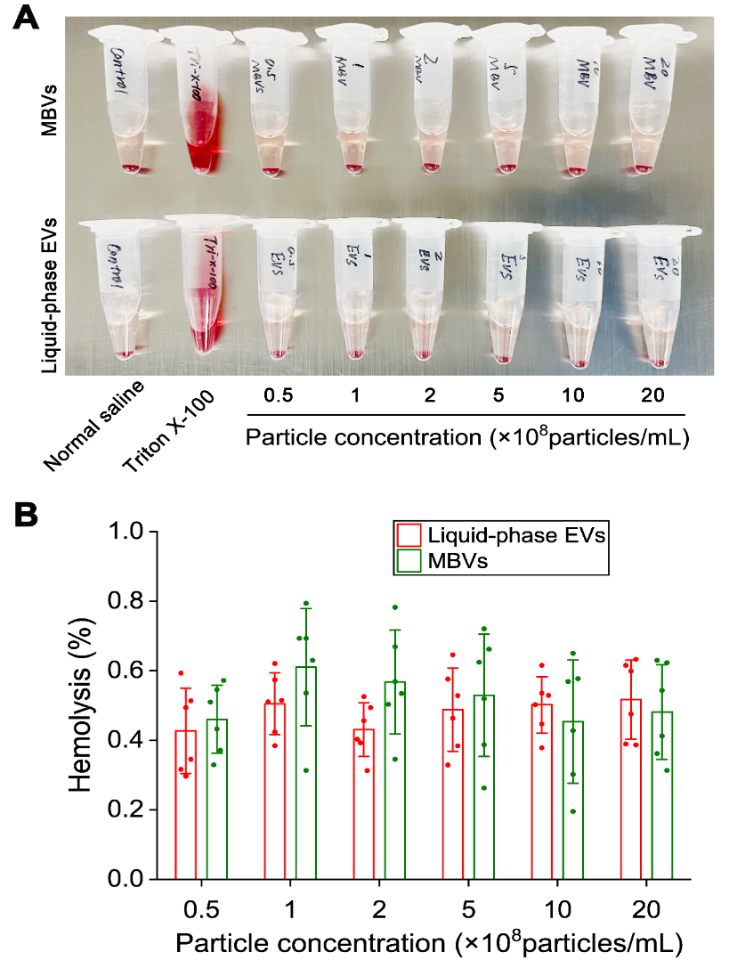


**Figure S1. Hemolytic assays for liquid-phase EVs and acellular tumor MBVs across varying concentration gradients.** (A) Representative images illustrating the effects of liquid-phase EVs and acellular tumor MBVs on a 2% red blood cell suspension, with normal saline serving as the negative control and Triton X-100 as the positive control. (B) Comparative analysis of hemolytic assay values for liquid-phase EVs and acellular tumor MBVs at different concentration gradients (n = 6).


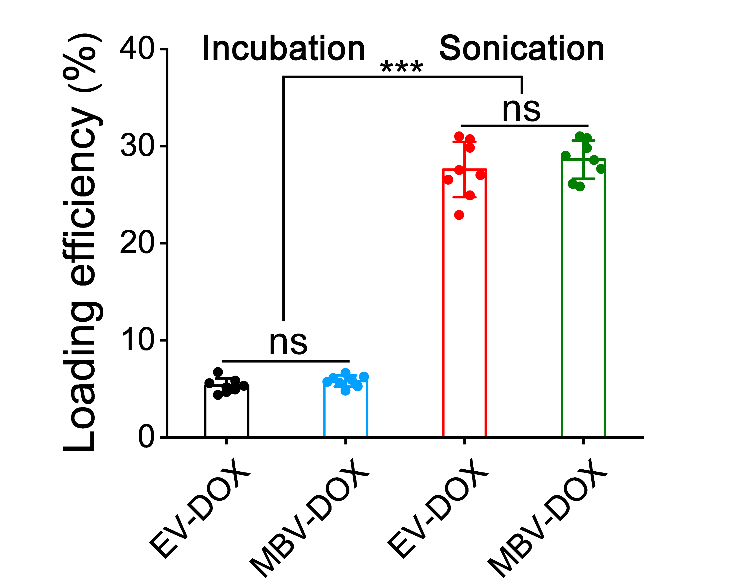


**Figure S2. The loading efficiency of doxorubicin (DOX) in both liquid-phase EVs and acellular tumor MBVs following incubation or sonication treatment (n = 6).** ****p* < 0.001 and ns, not significant.


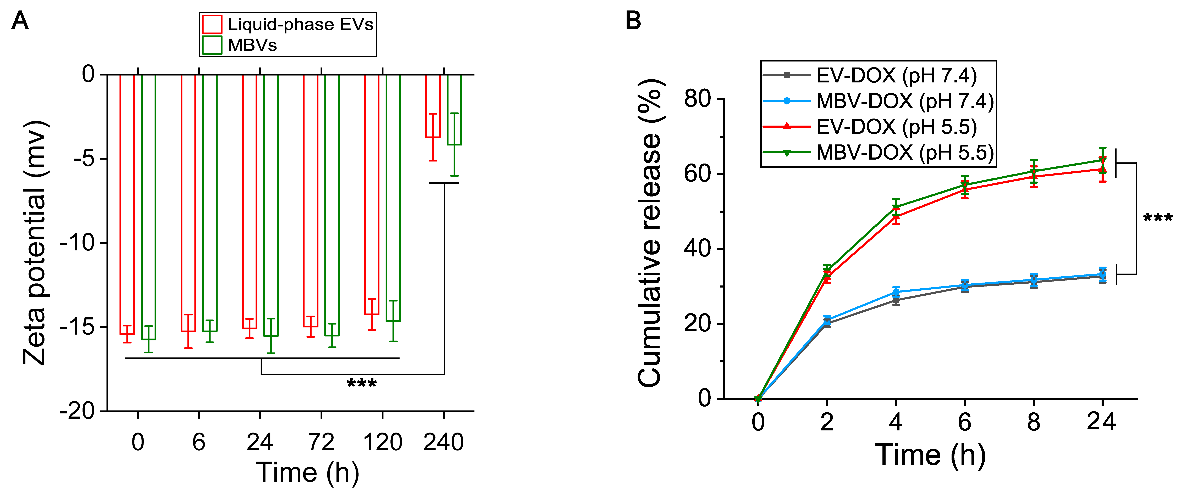


**Figure S3. The stability of the liquid-phase EVs and acellular tumor MBVs, as well as the drug release ability of EVs versus MBVs after loading DOX.** (A) The zeta potentials of liquid-phase EVs and acellular tumor MBVs stored at 4 °C with time (n = 3). (B)The *in vitro* release profiles of DOX from EV-DOX and MBV-DOX at pH values of 7.4 and 5.5 (n = 6). ****p* < 0.001.


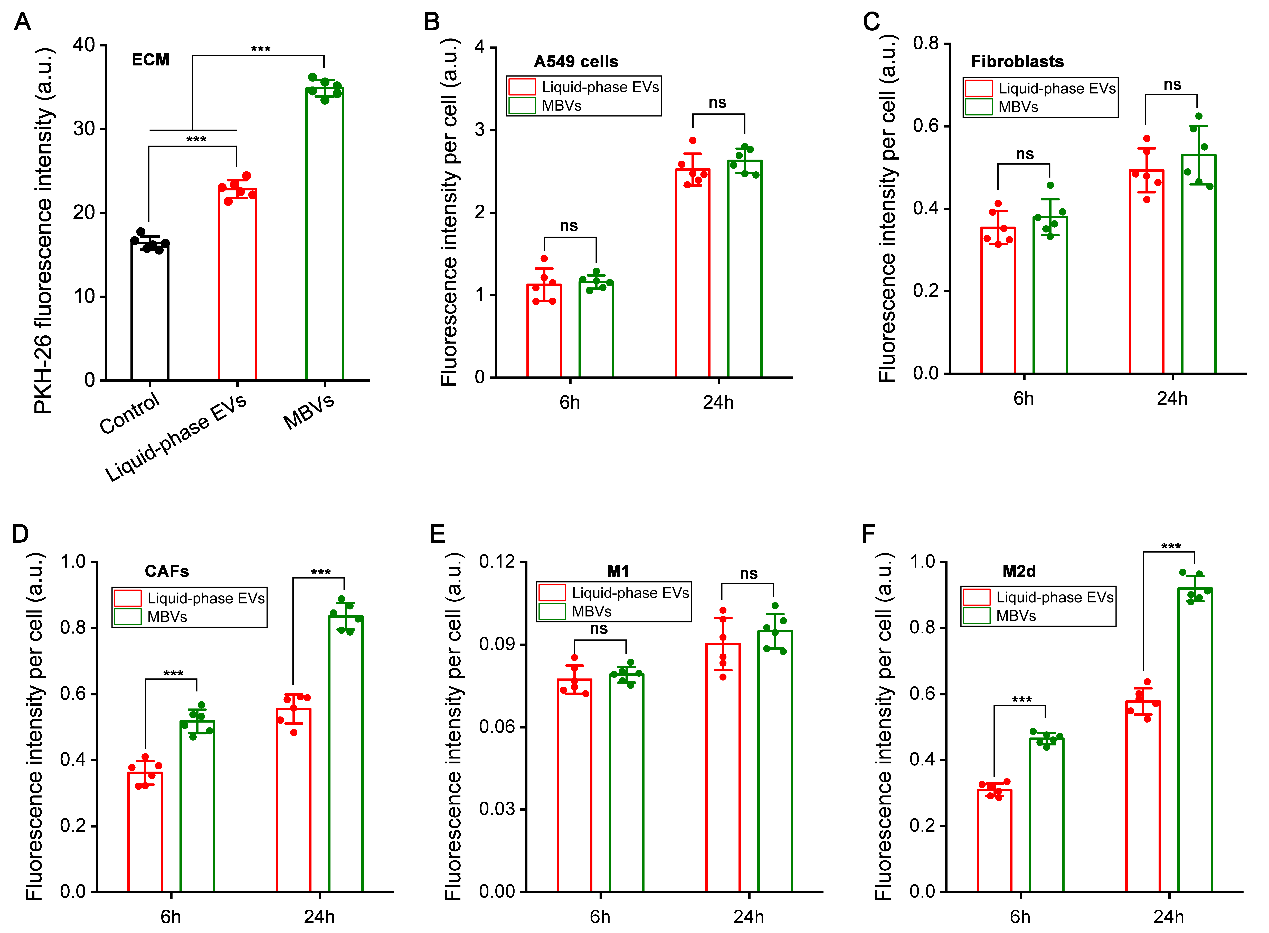


**Figure S4. Fluorescence intensity comparisons for MBV binding affinity (n = 6).** (A) The PKH-26 fluorescence intensity for both liquid-phase EVs and acellular tumor MBVs following incubation with decellularized tumor ECM. (B-F) The PKH-26 fluorescence intensity for liquid-phase EVs and acellular tumor MBVs after incubation with various cell types: A549 cells (B), fibroblasts (C), CAFs (D), M1 macrophages (E), and M2d macrophages (F). ****p* < 0.001 and ns, not significant.


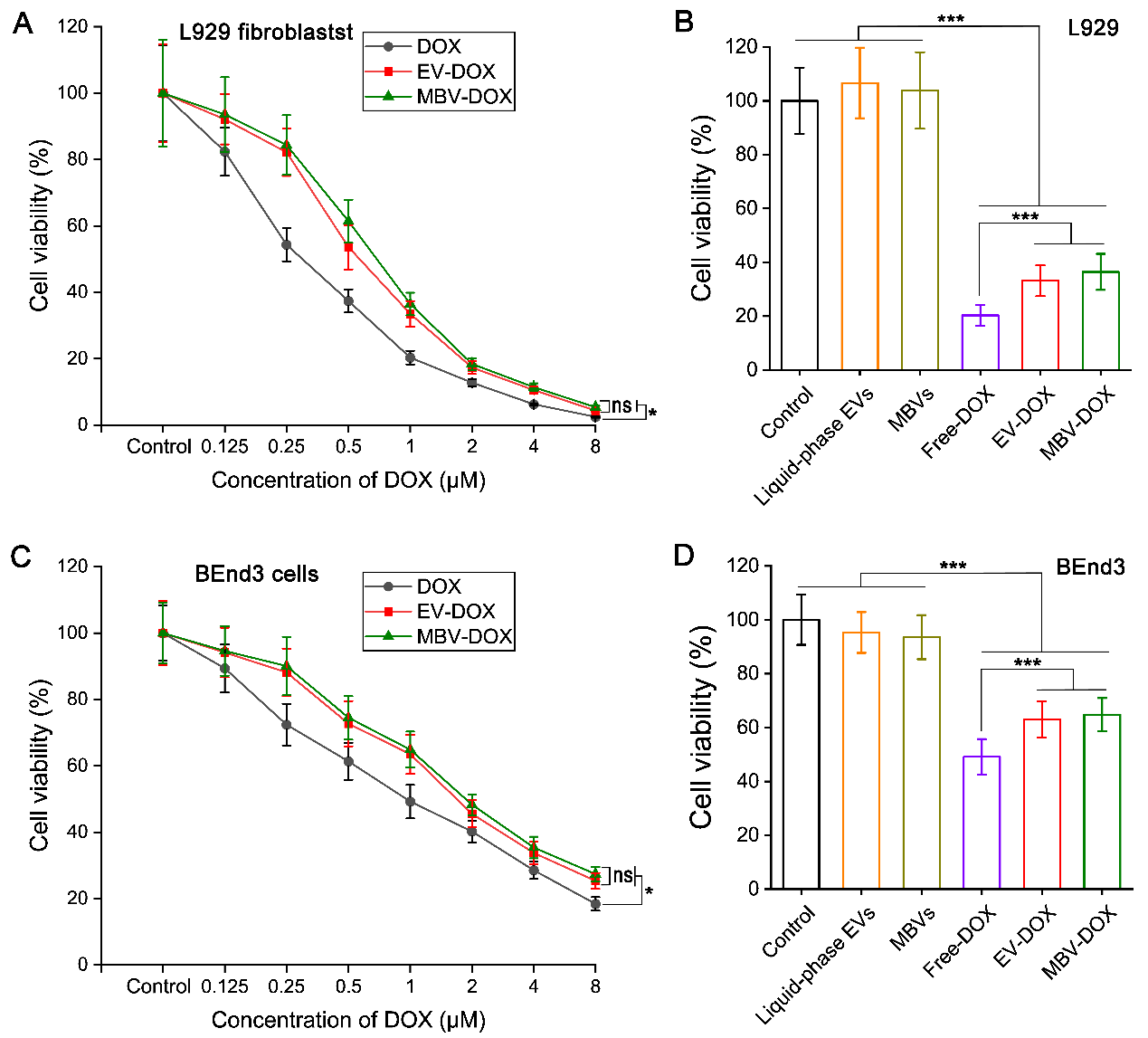


**Figure S5. The cytotoxic effects of MBVs and MBV-DOX on** **L929 mouse fibroblasts and mouse brain-derived Endothelial cells 3 (BEND3) assayed by CCK8.** (A, C) The cell viability of L929 and BEND3 cells following incubation with varying concentrations of DOX, EV-DOX, and MBV-DOX. (B, D) The cell viability of L929 and Bend3 cells after exposure to a PBS control, liquid-phase EVs, MBVs, DOX, EV-DOX, and MBV-DOX, with the DOX concentration fixed at 1.0 μM. n = 6, **p* < 0.05, ****p* < 0.001, and ns, not significant.


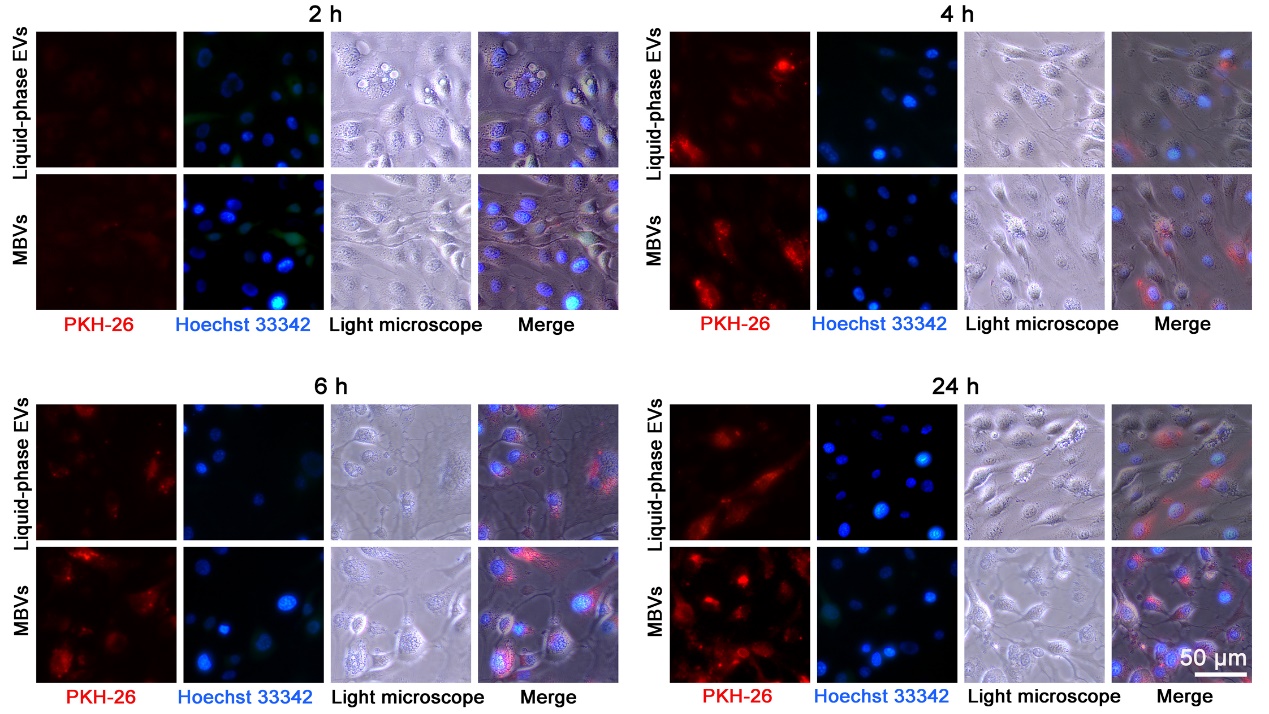


**Figure S6. The affinity of liquid-phase EVs and acellular tumor MBVs for CAFs over varying incubation periods.** The vesicles were labeled with PKH-26, and the nuclei were stained with Hoechst 33342.


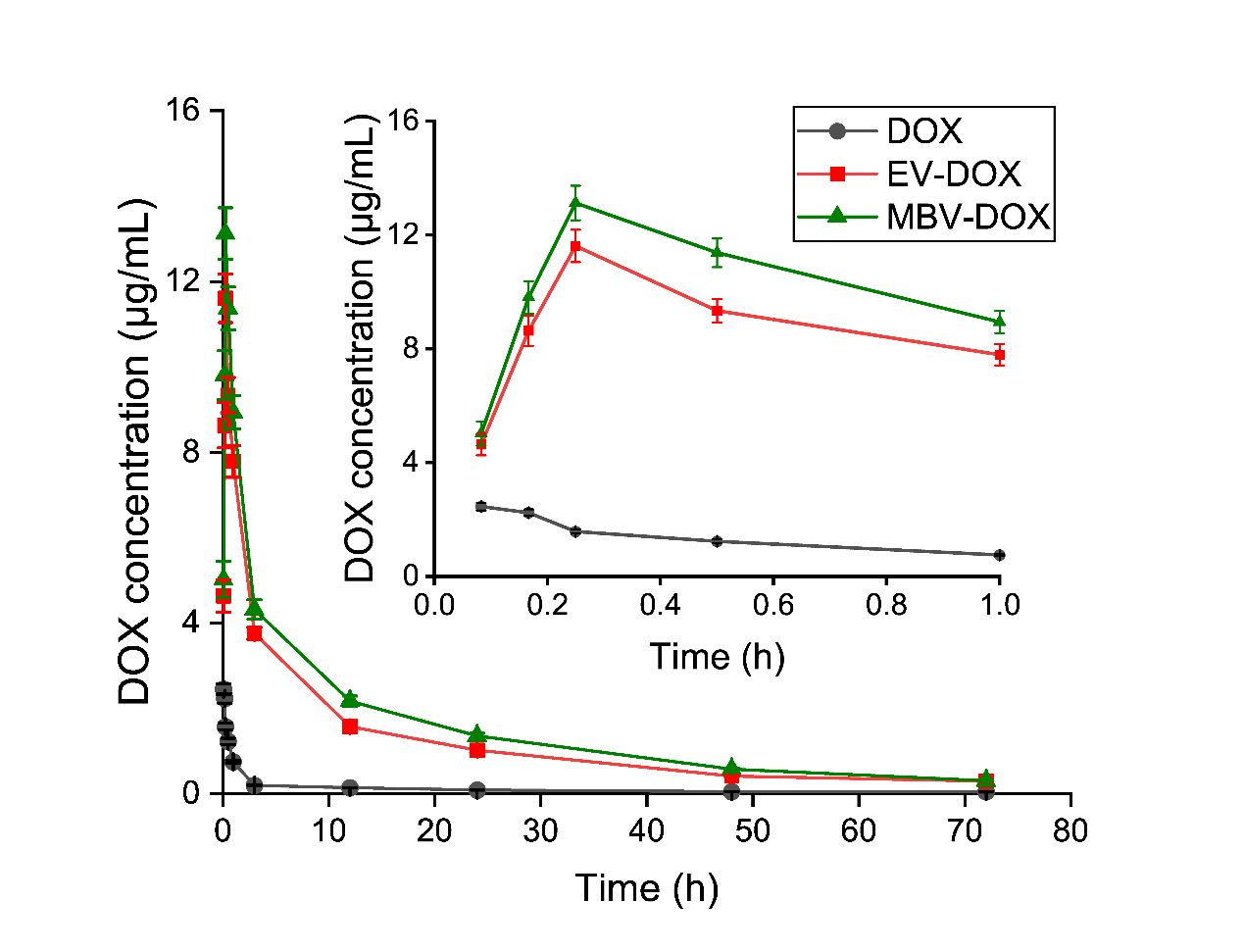
 **Figure S7. The concentration-time profiles of free DOX, EV-DOX, and MBV-DOX following the administration of a DOX dosage of 5 mg/kg.**

**Table S1. Plasma pharmacokinetic parameters following intravenous administration in SCID mice (n=6).**

| Parameter | Free-DOX | EV-DOX | MBV-DOX |
| --- | --- | --- | --- |
| C_max_ (μg/mL) | 2.45±0.13 | 11.61±0.57*** | 13.12±0.61***## |
| T_max_ (h) | 0.11±0.02 | 0.22±0.04*** | 0.23±0.04*** |
| t_1/2_ (h) | 0.56±0.06 | 2.14±0.24*** | 2.31±0.23*** |
| AUC_0-t_ (μg·h/mL) | 14.25±4.69 | 283.46±35.87*** | 313.67±41.13*** |

Abbreviations: C_max_, maximum drug concentration; T_max_, time to reach the observed maximum concentration; t_1/2_, elimination half-life; AUC_0-t_, area under concentration-time curve from 0 to last point. ****p* < 0.001 compared to the free-DOX group, ##*p* < 0.01 compared to the EV-DOX group.


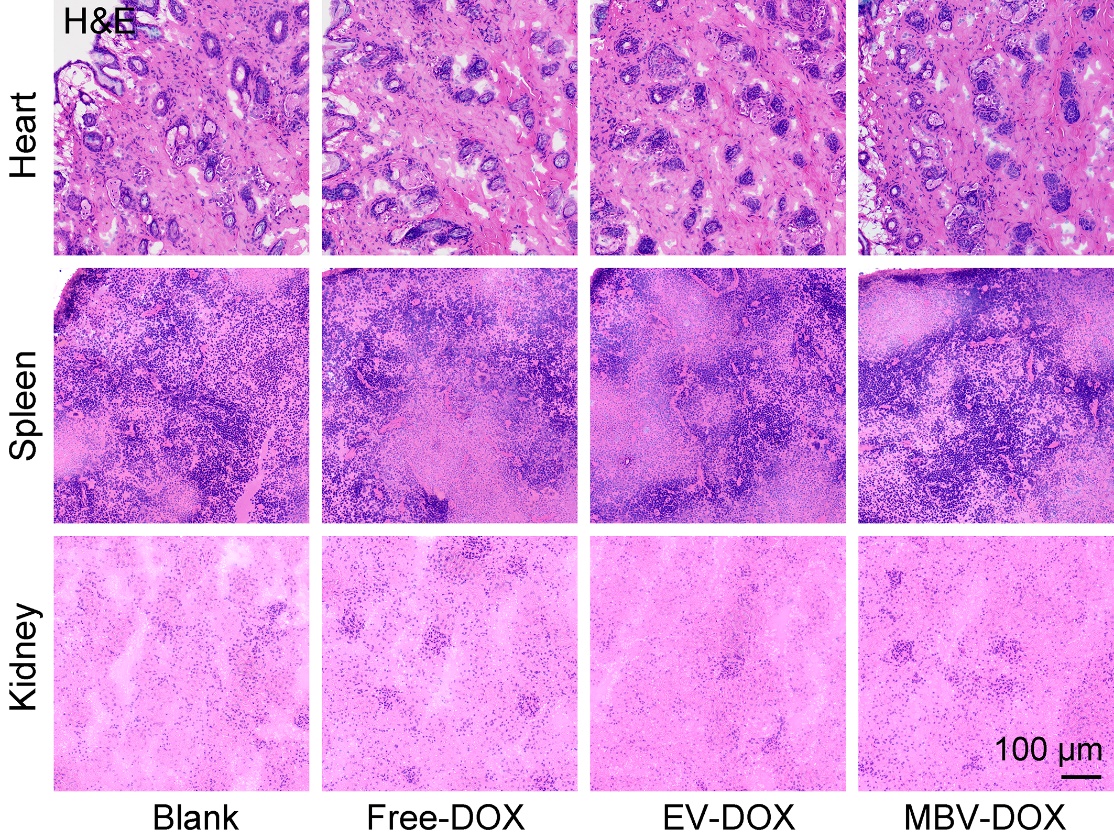


**Figure S8. H&E staining of heart, spleen, and kidney tissues from tumor-bearing mice treated with PBS, free DOX, EV-DOX, or MBV-DOX.** No significant differences were observed among the four treatment groups.


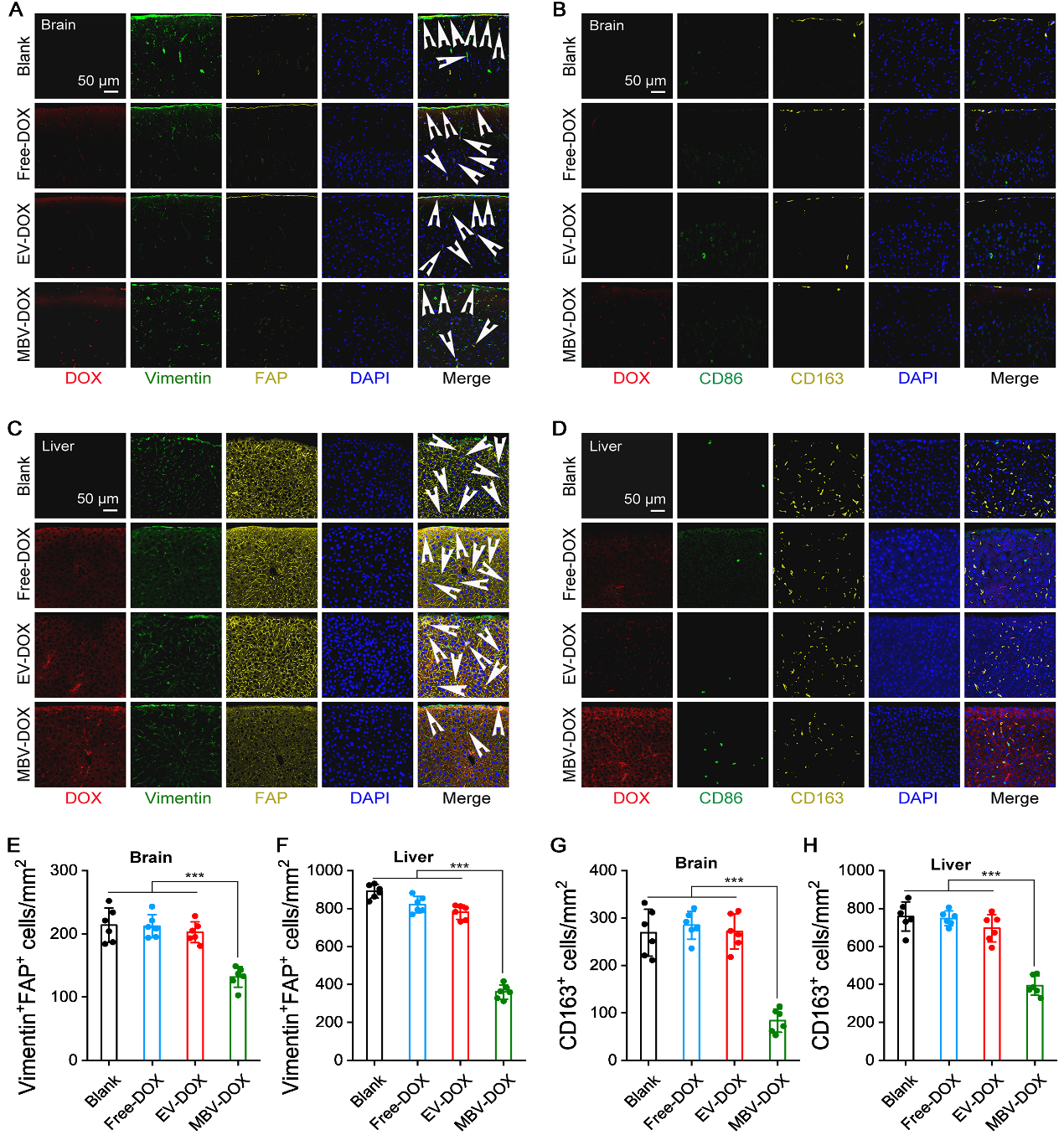


**Figure S9. Immunofluorescence staining of fibroblasts and macrophages in brain and liver tissues.** (A, B) Images of fibroblasts, CAFs (white arrows), and M1/M2 macrophages in the brain. (C, D) Liver images showing the same cell types. DOX-treated cells fluoresce red, and nuclei are blue. (E, F) Comparison of vimentin and FAP double positive cell numbers in brain and liver tissues. (G, H) Comparison of M2-like macrophage numbers in brain and liver. The MBV-DOX group showed the lowest cell numbers. ****p* < 0.001.


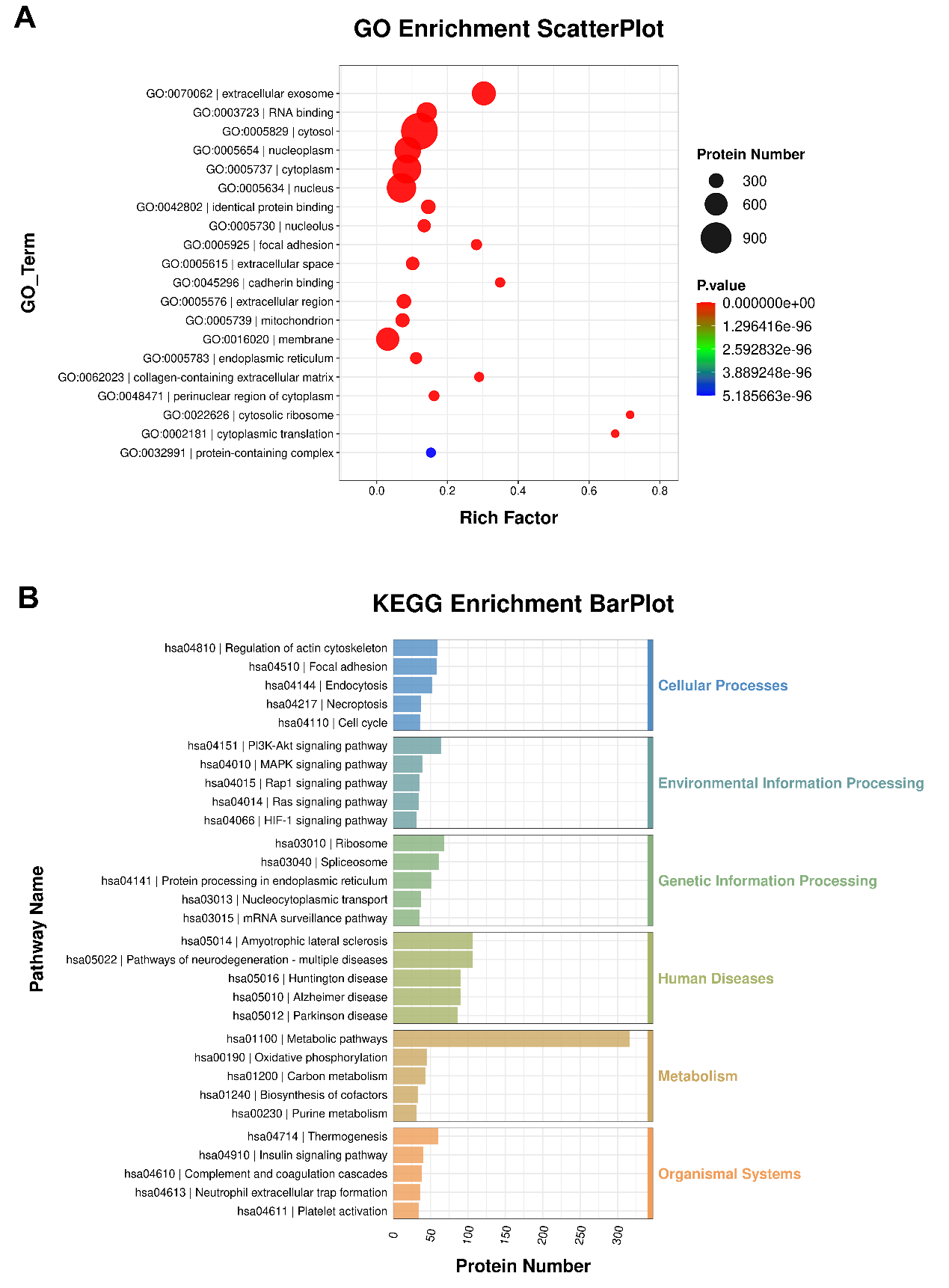


**Figure S10. Functional analysis of DEPs in acellular tumor MBVs and liquid-phase EVs.** (A) Analysis of the top 30 enriched GO terms among 2,247 DEPs. (B) Analysis of the top 20 enriched KEGG pathways associated with the DEPs. A larger value indicates a greater proportion of DEPs annotated to the respective pathway.
